# Integrative Network Pharmacology and Multi-Omics Analysis Reveal Key Targets and Mechanisms of Saikosaponin B1 Against Acute Lung Injury

**DOI:** 10.3390/metabo15120782

**Published:** 2025-12-04

**Authors:** Yuanfei Niu, Meiting Liu, Shuang Cui, Kaiyang Liu, Mengyuan Yang, Xiaozhen Hu, Changhui Zheng, Lianmei Wang, Junling Cao

**Affiliations:** 1 School of Chinese Materia Medica, Beijing University of Chinese Medicine, Beijing 102488, China; 20220941494@bucm.edu.cn (Y.N.); linhq1222@bucm.edu.cn (X.H.); 20220935066@bucm.edu.cn (C.Z.); 2 State Key Laboratory for Quality Ensurance and Sustainable Use of Dao-di Herbs, Institute of Chinese Materia Medica, China Academy of Chinese Medical Sciences, Beijing 100700, China; 20230931219@bucm.edu.cn (M.L.); 20190935109@bucm.edu.cn (S.C.); kyliu@icmm.ac.cn (K.L.); 20210941420@bucm.edu.cn (M.Y.)

**Keywords:** saikosaponin B1, acute lung injury, network pharmacology, multi-omics, molecular docking, molecular dynamics simulation

## Abstract

**Background/Objectives**: Acute lung injury (ALI) is a severe condition driven largely by inflammation and has limited therapeutic options. Although saikosaponin B1 (SSB1), a primary bioactive saponin from *Bupleurum* Radix, has demonstrated anti-inflammatory properties, its efficacy against ALI and its corresponding molecular mechanisms remain largely unexplored. This study employed an integrated approach combining network pharmacology, transcriptomics, and metabolomics to decipher the protective mechanisms of SSB1 against ALI. **Methods**: Potential targets were identified via network pharmacology, and core targets were validated through molecular docking, dynamics simulations, and independent GEO transcriptomic datasets. Experimental validation was performed in an LPS-induced murine ALI model, combining histopathology, ELISA, and integrated transcriptomic and metabolomic analyses. **Results**: Integrated analyses identified IL1B, TNF, and IL6 as core targets through which SSB1 exerts its anti-ALI effects. These targets were validated by high-affinity binding in simulations, confirmed in independent GEO transcriptomic datasets, and shown to be normalized by SSB1 treatment in vivo. Mechanistically, SSB1 appears to modulate the NOD-like receptor and cGAS-STING signaling pathways and rectify the key metabolic pathways orchestrated by these targets, including glycerophospholipid, arachidonic acid, and linoleic acid metabolism. **Conclusions**: This study systematically investigates the therapeutic effects of SSB1 against ALI by identifying its potential targets and underlying pathways. These results provide crucial mechanistic insights and robust experimental support, thereby paving the way for the clinical translation of SSB1.

## 1. Introduction

Acute lung injury (ALI) is a severe pulmonary disorder marked by diffuse alveolar damage, enhanced vascular permeability, pulmonary edema, and a robust inflammatory response. It carries substantial morbidity and mortality and may progress to acute respiratory distress syndrome (ARDS) [1,2]. The global outbreak of Coronavirus Disease 2019 (COVID-19) has highlighted ALI as a critical driver of severe ARDS, underscoring its significant threat to human health. Furthermore, ALI contributes to the pathogenesis of numerous chronic pulmonary conditions, including idiopathic pulmonary fibrosis, chronic obstructive pulmonary disease, asthma, and lung cancer [3]. Intense inflammatory responses and oxidative stress, driven particularly by severe cytokine storms, are central mechanisms in the pathogenesis of ALI [4]. Consequently, targeting these dysregulated processes represents a key therapeutic strategy for ALI management [5]. Current clinical management of ALI primarily involves lung-protective ventilation, pharmacotherapy including corticosteroids and inhaled pulmonary vasodilators, and supportive care such as extracorporeal membrane oxygenation, with the goal of reducing pulmonary inflammation and delaying disease progression [6]. However, the therapeutic efficacy of these strategies is often limited by side effects and the emergence of antibiotic resistance [7]. Consequently, mortality rates associated with ALI remain high, at approximately 40% [8]. Given the absence of specific therapies for this common critical illness, developing novel treatment strategies to improve therapeutic outcomes is crucial [9].

Traditional Chinese Medicine (TCM), characterized by its “holistic concept” and “treatment based on syndrome differentiation”, demonstrates distinct therapeutic potential and has garnered significant interest for managing acute pneumonia and COVID-19 [10]. The growing interest in TCM has spurred research into its derived interventions, such as active herbal ingredients and compound formulations, which have demonstrated considerable efficacy in models of ALI. A prominent example is *Bupleurum* Radix (BR), sourced from the roots of *Bupleurum chinense* DC. or *Bupleurum scorzonerifolium* Willd. First documented as a superior medicine in the “*Shennong Ben Cao Jing*”, this herb has been confirmed by modern pharmacological studies to possess significant anti-inflammatory activity, a property largely attributed to its rich saponin components [11]. Research on the anti-inflammatory effects of saikosaponins has centered predominantly on saikosaponin A (SSA) and saikosaponin D (SSD) [12,13]. Nevertheless, both SSA and SSD are hampered by their inherent instability and tendency to transform. In contrast, their isomer, saikosaponin B1 (SSB1), has recently emerged as a compound of interest due to its diverse pharmacological profile, which demonstrates anti-inflammatory, antiviral, anticancer, and hepatoprotective effects [14,15]. Given the central role of inflammation in the pathogenesis of ALI, these findings suggest its therapeutic potential. However, it remains unclear whether SSB1 protects against ALI and what its underlying anti-inflammatory mechanisms are.

As core disciplines of systems biology, network pharmacology, metabolomics, and transcriptomics establish a powerful framework for elucidating the multi-component, multi-target, and multi-pathway therapeutic nature of TCM. Specifically, network pharmacology elucidates complex biomolecular networks computationally [16]; metabolomics quantifies dynamic fluctuations in endogenous metabolites [17]; and transcriptomics delineates genome-wide transcriptional pathways [18,19]. The integration of these omics technologies overcomes the limitations of reductionist approaches, enabling a systematic investigation of TCM’s holistic mechanisms in complementary dimensions and thereby effectively bridging its philosophical “holistic view” with the paradigm of modern precision medicine. Given the multifactorial pathogenesis of ALI, an integrated analysis combining network pharmacology with multi-omics data is essential for systematically predicting the mechanisms by which SSB1 modulates the disease, offering a robust strategy to elucidate its complex interactions.

This study systematically investigated the therapeutic effects and protective mechanisms of SSB1 against ALI using an integrated approach that combines network pharmacology, transcriptomics, and metabolomics to identify its core targets and underlying pathways. Our findings offer novel insights into the therapeutic potential of SSB1, thereby laying a scientific foundation for its future development.

## 2. Materials and Methods

### 2.1. Identification of SSB1 Targets

The canonical SMILES, InChI, and structure-data file (SDF) of SSB1 were retrieved from PubChem. Its potential biological targets were systematically predicted using six public databases: ChEMBL, TargetNet, CTD, BATMAN-TCM, SwissTargetPrediction, and PharmMapper. The resulting targets were filtered and annotated against the *Homo sapiens* proteome (UniProt ID UP000005640) to select human targets. Detailed database URLs and access dates are provided in Supplementary Table S1.

### 2.2. Identification of ALI Targets

Disease-associated targets for ALI were systematically retrieved from four public databases: GeneCards, HERB, OpenTargets, and CTD. After standardization and deduplication, a unified set of potential ALI targets was established. The respective contributions of these databases were visualized with a Venn diagram. Subsequent intersection of this ALI target set with predicted SSB1 targets yielded candidate key targets for mechanistic investigation. Detailed retrieval parameters, including score thresholds and database access information, are provided in Supplementary Table S2.

### 2.3. Construction and Analysis of the SSB1–Target–ALI Network

To elucidate the relationships between SSB1, its targets, and ALI, an “SSB1–target–ALI” interaction network was constructed using Cytoscape. Nodes represent SSB1 and its target proteins, while edges denote interactions between them. The topological properties of the network were quantified, with node degree serving as the primary metric for evaluating node importance. Detailed information on the software version and analysis tools is provided in the Supplementary Methods.

### 2.4. PPI Network Analysis

To elucidate the mechanisms of SSB1–ALI targets, a protein–protein interaction (PPI) network was constructed. The common targets were submitted to the STRING database to obtain PPI data. The resulting interaction network was then imported into Cytoscape for visualization and refined by removing isolated nodes. Core targets within the network were identified as nodes where the degree, betweenness centrality (BC), and closeness centrality (CC) all exceeded their respective median values. Detailed database parameters and software information are provided in Supplementary Table S3.

### 2.5. GO and KEGG Pathway Enrichment Analyses

Enrichment analyses of Gene Ontology (GO) terms and Kyoto Encyclopedia of Genes and Genomes (KEGG) pathways were performed on the common targets of SSB1 and ALI using the clusterProfiler R package. Significantly enriched terms and pathways, filtered by statistical significance and relevance, were selected for visualization as bar and bubble plots, respectively. Detailed parameters, including the software version, significance thresholds, and number of top terms displayed, are cataloged in Supplementary Table S4.

### 2.6. MCODE Clustering Analysis

To elucidate functional relationships among the common targets, functional enrichment and module analysis were performed using the Metascape online platform. Functional modules were identified using the molecular complex detection (MCODE) algorithm to extract densely connected protein clusters. Additionally, potential governing transcription factors were predicted to infer regulatory relationships. Detailed platform access information and analysis parameters are provided in Supplementary Table S5.

### 2.7. Network Construction of Target–Pathway Interaction

The top enriched KEGG pathways for SSB1 and ALI were used to construct an “SSB1–target–pathway” network. To identify the final set of core therapeutic targets, nodes in this network were ranked by degree centrality. The top-ranked targets were integrated with the core targets previously identified from the PPI network. Detailed information on the number of pathways visualized and the software used is provided in the Supplementary Methods.

### 2.8. Molecular Docking

Molecular docking was performed to characterize the binding interactions between SSB1 and the core targets. The 3D structure of SSB1 was obtained from PubChem, while the crystal structures of the target proteins were sourced from the SCSB Protein Data Bank. The protein structures were prepared by removing water molecules, adding polar hydrogen atoms, and assigning partial charges. Docking simulations were conducted using AutoDock Vina by positioning SSB1 within the defined active site of each protein. The resulting binding poses were visualized to analyze binding modes and specific molecular interactions. Detailed software versions, database access information, and docking parameters are provided in Supplementary Table S6.

### 2.9. Molecular Dynamics

Molecular dynamics simulations were conducted for 100 ns on the docked complexes to assess their conformational stability. The system stability was evaluated by calculating the root mean square deviation (RMSD), root mean square fluctuation (RMSF), radius of gyration (Rg), solvent accessible surface area (SASA), and hydrogen bond numbers. The Gibbs free energy landscape was constructed as a function of RMSD and Rg to identify low-energy conformational states. Additionally, binding free energy was calculated using the MM/PBSA approach over the last 10 ns of the trajectory, with per-residue energy decomposition performed to identify key interacting residues. Complete simulation parameters and software details are provided in the Supplementary Methods.

### 2.10. Acquisition and Target Validation from the GEO Database

Two transcriptomic datasets (GSE2411 and GSE263867) related to ALI were retrieved from the GEO database for differential expression analysis. The GSE2411 dataset included 6 ALI and 6 control samples, while GSE263867 contained 5 ALI and 5 control samples. After log_2_ transformation and normalization to address batch effects, differential expression analysis was performed using distinct statistical thresholds tailored to each dataset’s technical characteristics. A threshold of |log_2_FC| > 0.585 and *p* < 0.05 was applied to GSE2411 (microarray data), while a more stringent cutoff of |log_2_FC| > 1 and *p* < 0.05 was used for GSE263867 (RNA-seq data) to account for their respective technical platforms and data properties. The results were visualized through heatmaps and volcano plots. The complete analytical workflow parameters are documented in Supplementary Table S7, and the rationale for the threshold selection is detailed in the Supplementary Methods.

### 2.11. Animals and Treatments

All experimental animals were obtained from Sibeifu (Beijing, China) Biotechnology Co., Ltd. (Certificate No. SCXK (Jing, China) 2024-0001). The study protocols were approved by the Research Ethics Committee of the Institute of Chinese Materia Medica, China Academy of Chinese Medical Sciences (Approval No.2024B152). All procedures were conducted in accordance with the National Institutes of Health (NIH) and ARRIVE guidelines for the care and use of laboratory animals.

Male *BALB/c* mice were acclimatized for 3 days and then randomly assigned to 5 groups (*n* = 6): (1) control (pure water i.g. + saline solution i.t.); (2) LPS (pure water i.g. + 3 mg/kg LPS i.t.); (3) LPS + DXMS (2 mg/kg i.g. + 3 mg/kg LPS i.t.); (4) LPS + SSB1-L (2.5 mg/kg); (5) LPS + SSB1-H (10 mg/kg i.g. + 3 mg/kg LPS i.t.). The doses of SSB1 (2.5 and 10 mg/kg) were selected to represent the low and high ends of the efficacious range, respectively, based on preliminary dose-finding studies. Treatment drugs were administered by oral gavage (0.1 mL/10 g) once daily for five consecutive days. Details on the dosing protocol and vehicle control rationale are in the Supplementary Methods.

Following a 5-day prophylactic treatment period, mice were fasted for 18 h with free access to water and then subjected to intratracheal instillation of LPS (3 mg/kg, 20 μL/10 g) to establish the ALI model. All mice were euthanized 24 h after the LPS challenge, and bronchoalveolar lavage fluid (BALF), serum, and lung tissue specimens were collected for subsequent analysis.

### 2.12. Reagents and Materials

LPS (L2630) was obtained from Sigma-Aldrich (St. Louis, MO, USA). SSB1 (JB268239) and Dexamethasone (DXMS, A301B224033), both with purities ≥98%, were obtained from Yuanye Bio-Technology Co., Ltd. (Shanghai, China). Murine ELISA kits for IL-6 (DY406-05), IL-1β (DY401-05), and TNF-α (DY410-05) were obtained from R&D SYSTEMS (Minneapolis, MN, USA).

### 2.13. H&E Staining

Lung tissues were fixed in 4% paraformaldehyde, embedded in paraffin using an automated tissue processor, and sectioned into 4 μm slices for hematoxylin and eosin (H&E) staining and subsequent histological examination.

### 2.14. Lung Wet/Dry (W/D) Weight Ratio

The right middle lung lobe was excised after euthanasia, rinsed with saline, blotted dry, and immediately weighed to obtain the wet weight (W). It was then dried at 65 °C until constant weight (typically 48 h) to determine the dry weight (D).

### 2.15. White Blood Cell Count

The cell pellet from the BALF was resuspended in 50 μL of phosphate-buffered saline (PBS), and a 10 μL aliquot was loaded onto a hemocytometer to enumerate the total leukocytes.

### 2.16. Measurement of BALF

The concentrations of IL-6, IL-1β, and TNF-α in undiluted mouse BALF samples were quantified using commercial ELISA kits (IL-6: DY406-05; IL-1β: DY401-05; TNF-α: DY410-05) following the manufacturers’ protocols.

### 2.17. Transcriptome

Total RNA was extracted from lung tissues for transcriptome sequencing. Sequencing libraries were constructed from high-quality RNA and sequenced on the Illumina NovaSeq X Plus platform with 150 bp paired-end reads. For bioinformatic analysis, raw reads underwent quality control and adapter trimming, followed by alignment to the mouse reference genome GRCm39. Transcript abundance was quantified, and differential gene expression analysis was performed with significance thresholds set at |log_2_FC| ≥ 1 and a false discovery rate (FDR) < 0.05. Functional enrichment analysis of GO terms and KEGG pathways was subsequently conducted on the DEGs. Detailed parameters and bioinformatics tools are listed in Supplementary Table S8.

### 2.18. Untargeted Metabolomic Analysis

Metabolite profiling was performed on lung tissue samples. Following homogenization in aqueous methanol and centrifugation, the supernatant was analyzed using a UHPLC–high–resolution mass spectrometry system. Metabolites were annotated by searching relevant databases, and data processing included quality control filtering, normalization, and log_10_ transformation. Principal component analysis (PCA) was employed to assess system stability and identify significant metabolites based on variable importance in projection (VIP > 1) and statistical significance (*p* < 0.05). Pathway enrichment analysis of significant metabolites was subsequently performed using the KEGG database. Detailed chromatographic conditions, mass spectrometry parameters, and database information are provided in Supplementary Table S9.

### 2.19. Determination of Arachidonic Acid Metabolite Content

Lipids were extracted from lung tissues using a cold methanol/acetonitrile solvent system supplemented with the antioxidant butylated hydroxytoluene (BHT). Following homogenization, sonication, and centrifugation at 4 °C, the supernatant was collected, concentrated under a nitrogen stream, and reconstituted for LC-MS/MS analysis. Chromatographic separation was achieved on a reversed-phase C18 column with an optimized gradient. Mass spectrometric detection was conducted using electrospray ionization in negative ion mode with multiple reaction monitoring (MRM). The comprehensive protocols for extraction, chromatography, and mass spectrometry are documented in Supplementary Table S10.

### 2.20. Statistical Analysis

Statistical analyses were conducted using GraphPad Prism (version 9.0, GraphPad Software, LLC, San Diego, CA, USA). Data are expressed as the mean ± standard error of the mean (SEM). Differences among multiple groups were assessed by one-way analysis of variance (ANOVA), followed by Tukey’s post hoc test for multiple comparisons. A *p*-value of less than 0.05 was considered statistically significant.

## 3. Results

### 3.1. Identification of SSB1 Targets

Integration of target prediction databases identified 206 unique potential targets for the active compounds (Supplementary Figure S1).

### 3.2. Prediction of Potential Therapeutic Targets for ALI

**Figure 1:**
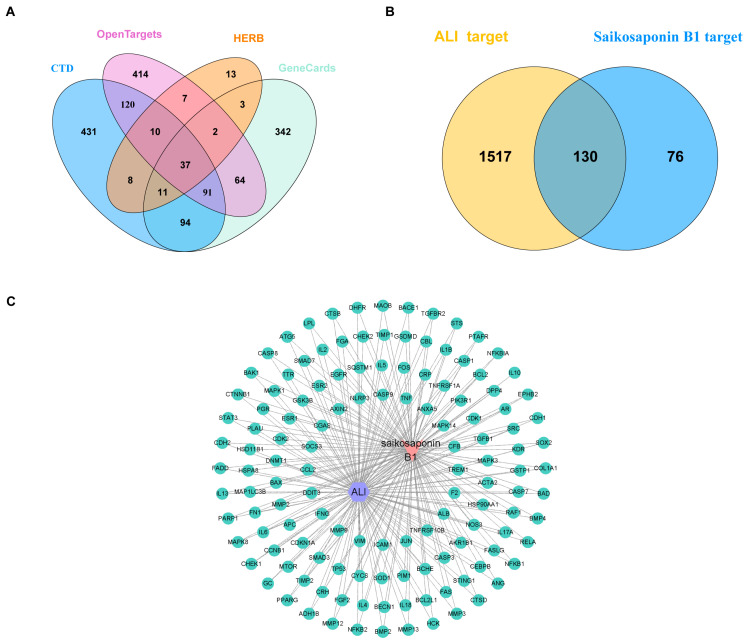
Identification of saikosaponin B1 (SSB1) and acute lung injury (ALI) targets and construction of the SSB1–ALI interaction network. (**A**) Intersection of ALI targets from multiple databases. (**B**) Intersection of SSB1 and ALI targets. (**C**) Construction of the SSB1–target–ALI network.

An intersection analysis between 1647 ALI-related targets (Figure 1A) and 206 SSB1 targets yielded 130 common targets (Figure 1B), highlighting them as potential mediators of the therapeutic effect of SSB1 against ALI.

### 3.3. Construction of the SSB1–Target–ALI Network

Topological analysis of the “SSB1–target–ALI” network revealed that SSB1 concurrently interacts with multiple targets, suggesting a synergistic multi-target mechanism against ALI (Figure 1C).

### 3.4. PPI Network Analysis

**Figure 2:**
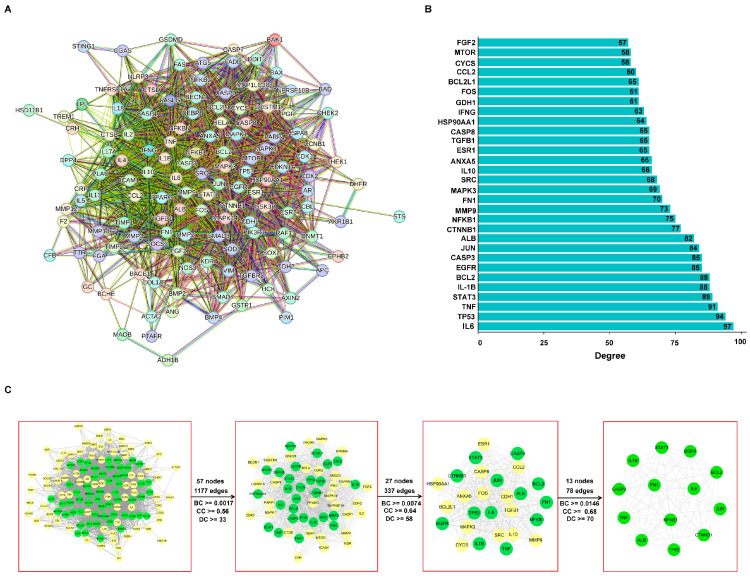
Analysis of the protein–protein interaction (PPI) network for common targets. (**A**) Construction of the PPI network from the STRING database. (**B**) Top 20 high-degree nodes within the PPI network. (**C**) Hub targets identified from the PPI network topology.

PPI network analysis of the 130 common targets first identified 20 key proteins including IL6, TP53, TNF, STAT3, IL1B, BCL2, EGFR, and CASP3. Subsequent topological screening (degree, BC, CC) refined this set to 13 core targets, including IL6, TP53, TNF, STAT3, and IL1B, thereby underscoring their pivotal role in SSB1’s action against ALI (Figure 2A–C).

### 3.5. GO Function and KEGG Pathway Enrichment Analysis

**Figure 3:**
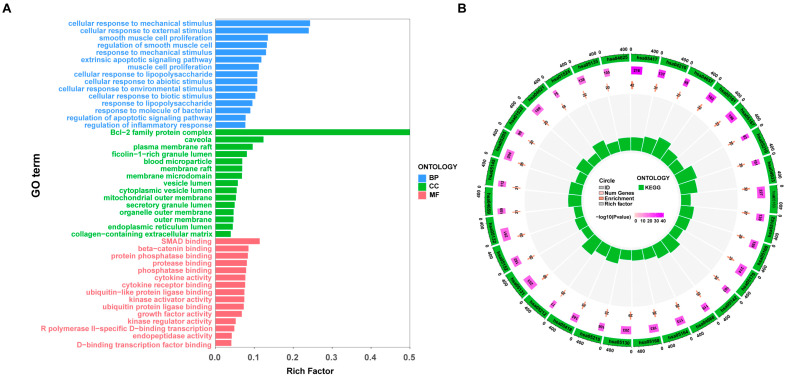
Gene Ontology (GO) and Kyoto Encyclopedia of Genes and Genomes (KEGG) enrichment analysis. (**A**) Top 15 enriched GO terms. (**B**) Top 30 enriched KEGG pathways.

GO and KEGG enrichment analyses of the 130 common targets elucidated the multi-faceted mechanisms of SSB1 against ALI. Significant terms included the cellular response to LPS (biological process, BP), SMAD and β-catenin binding (molecular function, MF), and caveolae (cellular component, CC). KEGG analysis highlighted the NOD-like receptor, IL-17, and TNF signaling pathways (Figure 3A,B). Collectively, these pathways form a synergistic network regulating core ALI pathologies, inflammatory response, cell survival, and angiogenesis, thereby elucidating the multi-pathway therapeutic mechanism of SSB1. Key targets within the NOD-like receptor signaling pathway (hsa04621) relevant to ALI are shown in Supplementary Figure S2.

### 3.6. MCODE Clustering Analysis

**Figure 4:**
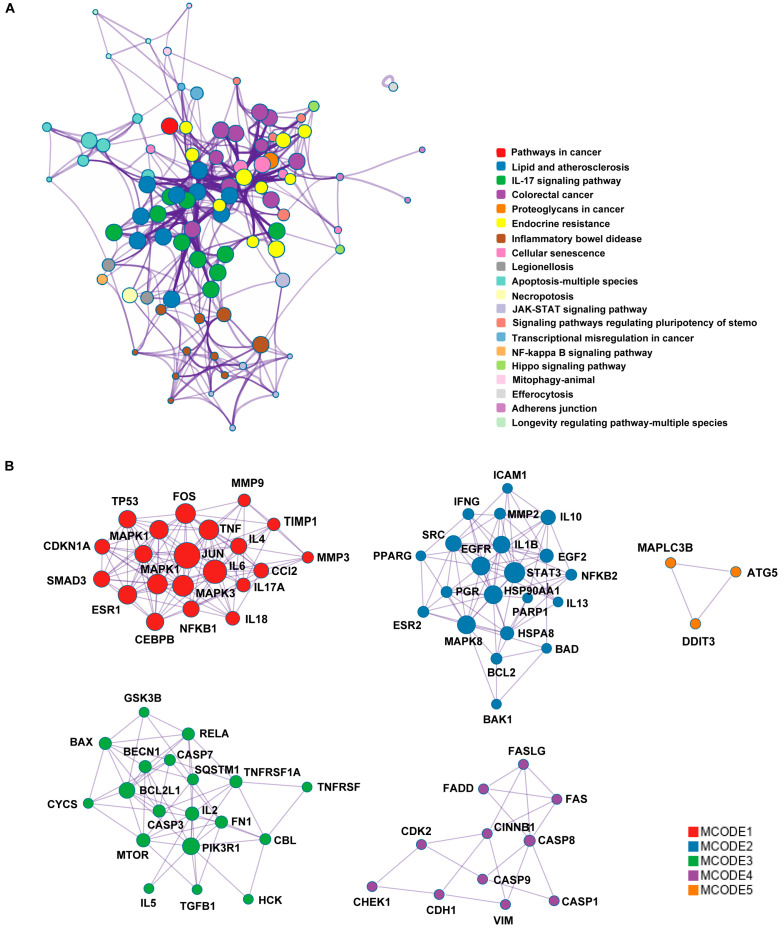
Modular analysis of the protein–target network for SSB1 in ALI. (**A**) PPI subnetwork of hub targets. (**B**) Molecular complex detection (MCODE) cluster analysis identifying functional modules.

Metascape analysis of the 130 common targets revealed significant enrichment in the IL-17, JAK-STAT, and NF-κB signaling pathways (Supplementary Figure S3). A target–pathway interaction network identified central hubs, including TNF, IL1B, IL6, TP53, MAPK1, CASP3, and STAT3, mediating crosstalk between functional clusters (Figure 4A). MCODE analysis further resolved five densely interconnected modules encompassing apoptosis regulators (BCL2, CASP8, FAS, and MAPK8) and inflammatory mediators (TNF, IL1B, IL6, IL10, and MMP2), demonstrating that SSB1 ameliorates ALI through a multi-target mechanism converging on immune regulation, inflammatory signaling, and cell fate decisions (Figure 4B).

### 3.7. Construction of the Target–Pathway Network and Identification of Core Targets

**Figure 5:**
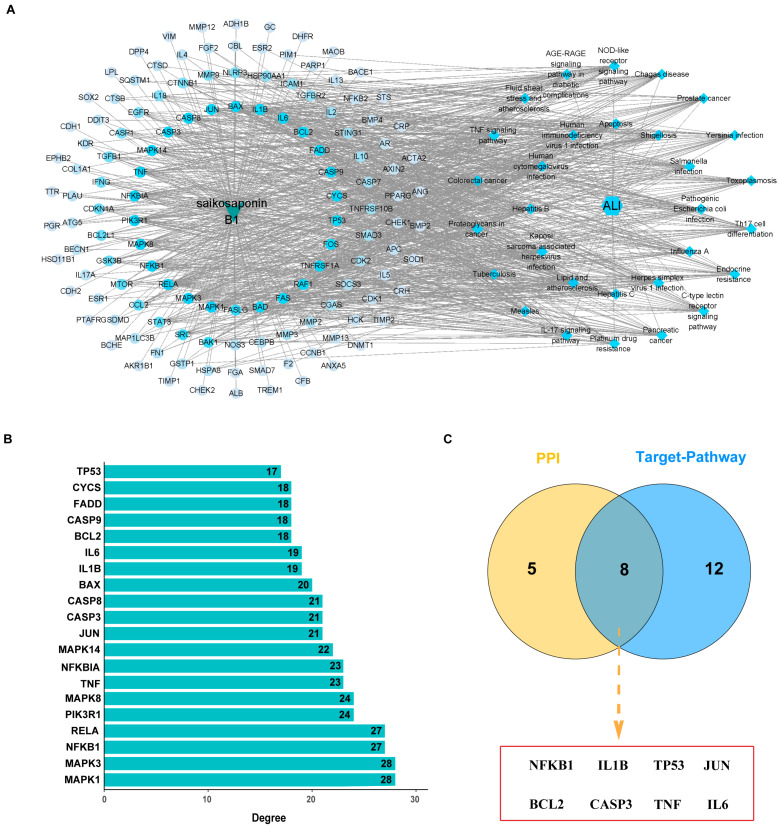
Target–pathway network construction and core target identification. (**A**) The “SSB1–target–pathway–ALI” interaction network. (**B**) Top 20 targets ranked by degree centrality. (**C**) Overlap of core targets from PPI and network analyses.

Construction of an “SSB1–target–pathway–ALI” network demonstrated that the therapeutic effect is mediated by the concurrent modulation of multiple hub targets and interconnected pathways, rather than isolated pathway regulation (Figure 5A). Topological analysis identified 20 candidate targets, and subsequent intersection with the core PPI network confirmed 8 shared hubs as central to the therapeutic mechanism (Figure 5B,C).

### 3.8. Molecular Docking

**Figure 6:**
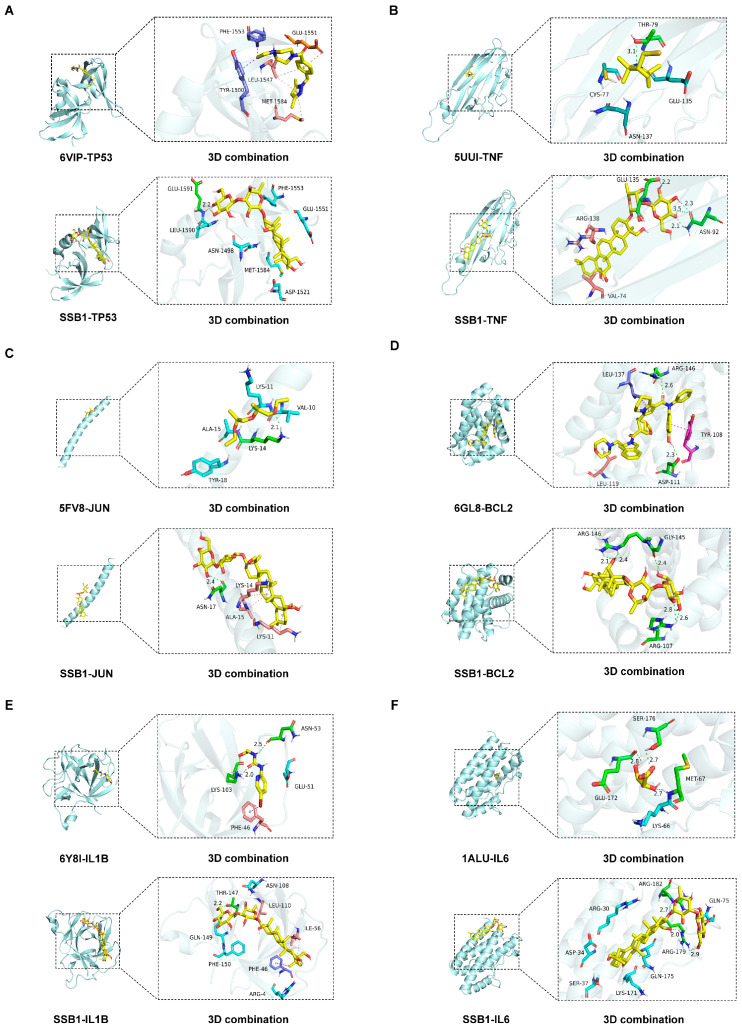
Molecular docking results confirming the binding of reference ligands and SSB1 to core targets. Detailed binding poses are shown for (**A**) TP53, (**B**) TNF, (**C**) JUN, (**D**) BCL2, (**E**) IL1B, and (**F**) IL6.

Molecular docking analysis demonstrated that SSB1 effectively binds to six core targets (TP53, TNF, JUN, BCL2, IL1B, and IL6). The binding free energies (ΔG) for all complexes were lower than −5.0 kcal/mol and more favorable than those of reference ligands (Supplementary Methods and Figure 6), indicating stronger binding affinity. Structural insights further revealed that SSB1 not only retained key interactions with active-site residues but also established additional molecular contacts. These results highlight SSB1 as a promising multi-target inhibitor with enhanced binding properties.

### 3.9. Molecular Dynamics Simulations

**Figure 7:**
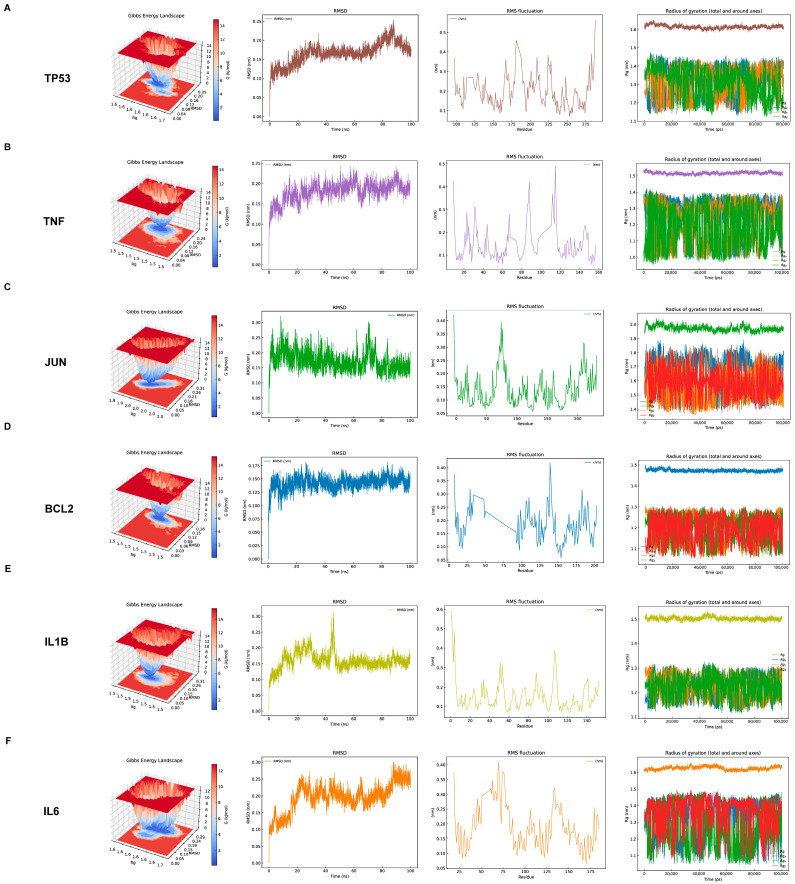
Molecular dynamics simulation results confirming the stability of SSB1–core target complexes. Representative simulation results for the complexes with (**A**) TP53, (**B**) TNF, (**C**) JUN, (**D**) BCL2, (**E**) IL1B, and (**F**) IL6.

Molecular dynamics simulations (100 ns) demonstrated stable binding of SSB1 to all core targets (TP53, TNF, JUN, BCL2, IL1B, and IL6) under physiological conditions (Figure 7A–F). All systems reached equilibrium with backbone RMSD fluctuations below 0.2 nm, and low RMSF values indicated minimal residual flexibility. Additionally, the hydrogen bonds and solvent-accessible surface of the complexes are shown in Supplementary Figure S4.

The SSB1–target complexes (TP53, TNF, JUN, BCL2, IL1B, and IL6) exhibited robust structural stability throughout the simulations. RMSD analysis confirmed that all systems rapidly reached equilibrium and maintained stable fluctuations around 0.2 nm. Corresponding RMSF profiles indicated moderate fluctuations (0.1–0.3 nm) across most residues, consistent with overall conformational stability, while several regions displayed elevated flexibility, potentially corresponding to functional loops or domains. Furthermore, stable Rg values affirmed the consistent compactness of all complexes.

### 3.10. Identifying and Validating Therapeutic Targets for ALI Using GEO Datasets

**Figure 8:**
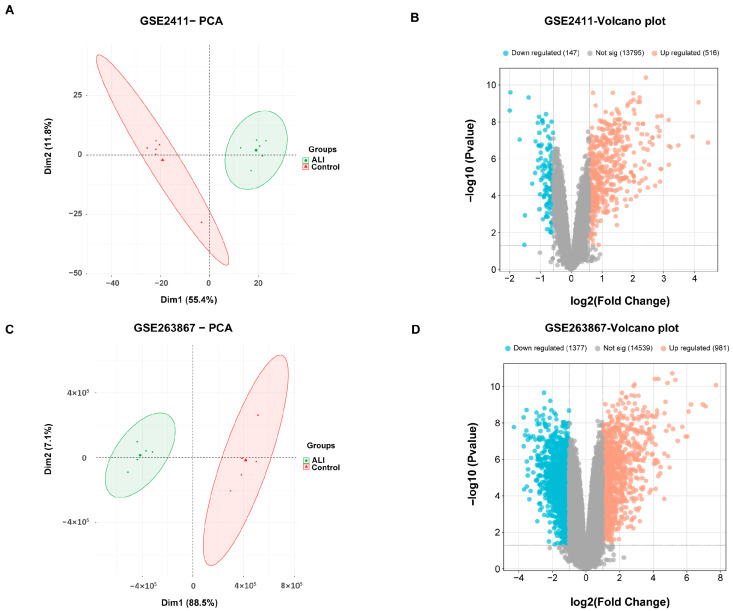
Analysis of ALI-related transcriptomic profiles from GEO datasets. (**A**) Principal component analysis (PCA) and (**B**) volcano plot displaying transcriptomic differences between control and ALI groups in the GSE2411 dataset. (**C**) PCA and (**D**) volcano plot for the GSE263867 dataset.

Following normalization of the expression matrices from the GEO database, differential expression analysis was performed on the datasets GSE2411 and GSE263867. GSE2411 contained 663 DEGs, comprising 516 upregulated and 147 downregulated genes, while GSE263867 contained 2358 DEGs, comprising 981 upregulated and 1377 downregulated genes. Sample distribution and gene expression patterns were visualized through PCA and heatmaps, respectively (Figure 8A–D), with the latter displaying the top 50 DEGs from each dataset (Supplementary Figure S5).

**Figure 9:**
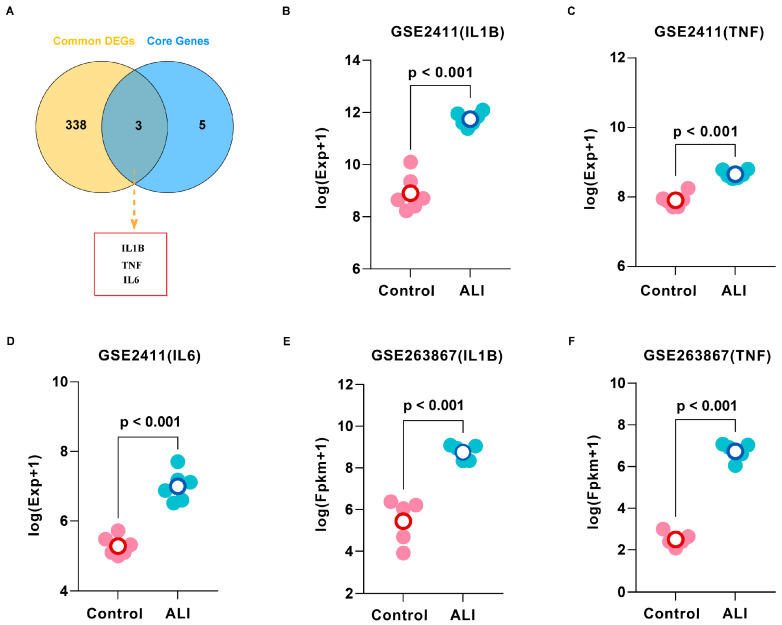
Identification and validation of core targets for ALI using GEO datasets. (**A**) Intersection of common differentially expressed genes (DEGs) and core targets from network pharmacology. (**B**) Validation of IL1B, (**C**) TNF, and (**D**) IL6 expression in the GSE2411 dataset; *n* = 6. (**E**) Validation of IL1B and (**F**) TNF expression in the GSE263867 dataset; *n* = 5.

To independently validate the eight core targets from the “SSB1–target–pathway” network, we analyzed the overlapping DEGs between the GSE2411 and GSE263867 datasets (Supplementary Figure S6A,B). Intersection of these DEGs with the core targets identified three common candidates: IL1B, TNF, and IL6 (Figure 9A). These key targets were significantly dysregulated in ALI samples across both datasets (Figure 9B–F), confirming their crucial roles in ALI pathogenesis at the transcriptomic level.

### 3.11. SSB1 Alleviates LPS-Induced ALI in Mice

**Figure 10:**
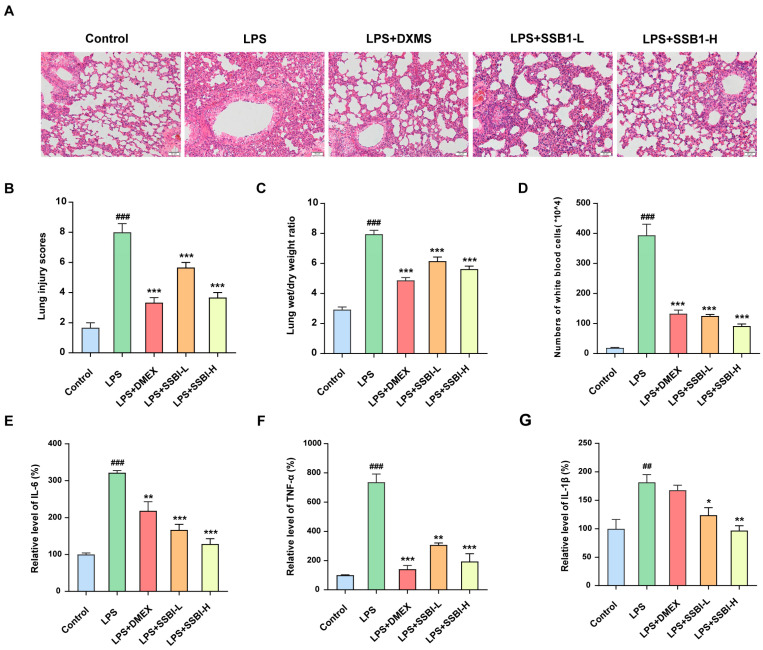
SSB1 alleviates LPS-induced ALI in mice. (**A**) Representative hematoxylin and eosin (H&E)-stained images of mouse lung section; scale bar = 50 μm. (**B**) Pathology scores of lung tissue; *n* = 6. (**C**) Measurement of lung wet/dry (W/D) weight ratio. (**D**) Number of white blood cells in bronchoalveolar lavage fluid (BALF). (**E**) Relative levels of IL-6, (**F**) TNF-α, and (**G**) IL-1β (normalized to the control group) in BALF. *n* = 6. **^##^** *p* < 0.01, **^###^** *p* < 0.001 vs. the control group. * *p* < 0.05, ** *p* < 0.01, and *** *p* < 0.001 vs. the LPS group.

LPS challenge successfully induced ALI in mice, as confirmed by characteristic pulmonary damage, elevated lung injury scores, increased lung W/D weight ratios, white blood cell counts, and pro-inflammatory cytokine levels. SSB1 treatment significantly ameliorated ALI, as evidenced by markedly reduced lung injury scores, decreased lung W/D weight ratios, and lower white blood cell counts (Figure 10A–G). Moreover, SSB1 administration potently and dose-dependently suppressed the levels of pro-inflammatory cytokines in BALF. Specifically, the low- and high-dose groups reduced IL-6 by 48.2% and 60.1%, TNF-α by 58.2% and 73.8%, and IL-1β by 31.8% and 46.8%, respectively (Figure 10E–G). Collectively, these results demonstrate that SSB1 protects against ALI largely through the suppression of pro-inflammatory cytokine release.

### 3.12. Transcriptome Analysis of SSB1 Against ALI

**Figure 11:**
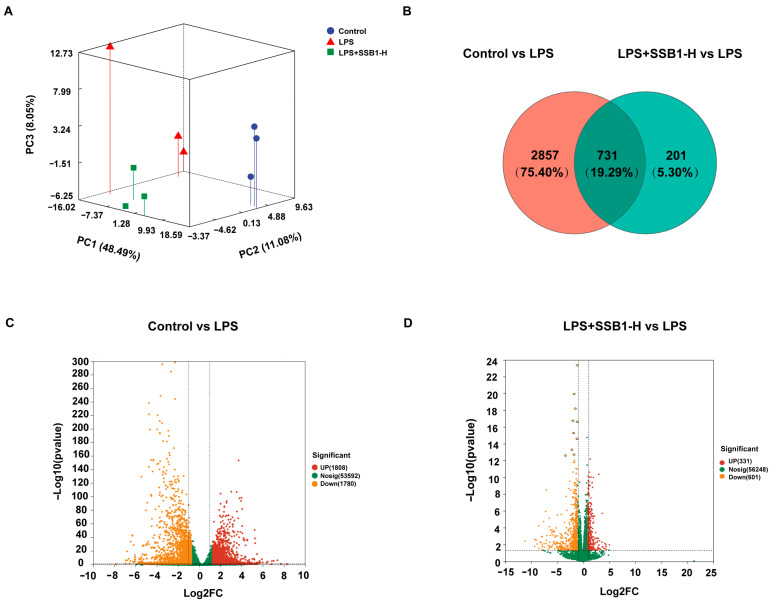
Transcriptome analysis of ALI lung tissue after SSB1 treatment. (**A**) PCA of LPS/LPS + SSB1-H group in positive and negative ion modes. (**B**) Changed genes of control vs. LPS and LPS + SSB1-H vs. LPS using Venn analysis. (**C**,**D**) Volcano plots of control vs. LPS, and LPS + SSB1-H vs. LPS, indicating upregulation (red), downregulation (orange), or no change (green) for different metabolites.

Transcriptomic profiling revealed distinct separation among experimental groups by PCA (Figure 11A). Differential expression analysis (*p* < 0.05, VIP > 1) identified 1780 up- and 1808 downregulated genes between the control and LPS groups (Figure 11C). In contrast, the LPS + SSB1-H group showed 331 upregulated and 601 downregulated genes relative to the LPS group (Figure 11D). Notably, we identified 731 genes that were significantly dysregulated by LPS (FDR < 0.05 and |log_2_FC| ≥ 1) and whose expression was significantly reversed by SSB1 treatment (Figure 11B). Functional annotation showed significant enrichment in immune processes including immune system activity, response to stimuli, and stress response (Supplementary Figure S7A). KEGG analysis highlighted significant involvement of the NOD-like receptor, Toll-like receptor, and IL-17 signaling pathways (Supplementary Figure S7B), supporting SSB1’s multi-target mechanism against ALI through the coordinated regulation of inflammatory pathways.

**Figure 12:**
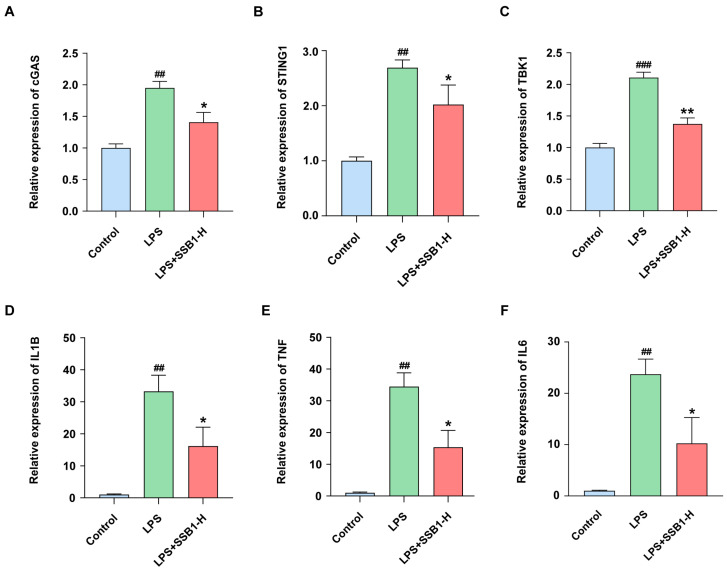
Key genes in lung tissue of SSB1 against ALI. (**A**–**C**) Relative expressions of three genes from cGAS-STING signaling pathway (normalized to the control group). (**D**–**F**) Relative expressions of three key genes from network pharmacology results (normalized to the control group); *n* = 3. **^##^** *p* < 0.01, **^###^** *p* < 0.001 vs. the control group. * *p* < 0.05, ** *p* < 0.01 vs. the LPS group.

Building on evidence that regulators like NLRP6, NOD1, NOD2, and NLRP12 interface with the cGAS-STING pathway [20], we found that SSB1 treatment significantly suppressed LPS-induced upregulation of key pathway components. Specifically, SSB1 reduced the expression of cGAS, STING, and TBK1 by 28.0%, 24.9%, and 34.8%, respectively (*p* < 0.05, *p* < 0.05, and *p* < 0.01, Figure 12A–C). Consistent with this anti-inflammatory activity, SSB1 concurrently reversed the LPS-induced upregulation of the core network targets IL1B, TNF, and IL6 by 51.4%, 55.4%, and 56.8%, respectively (*p* < 0.05, Figure 12D–F).

### 3.13. Metabolome Analysis of ALI Lung Tissue After SSB1 Treatment

**Figure 13:**
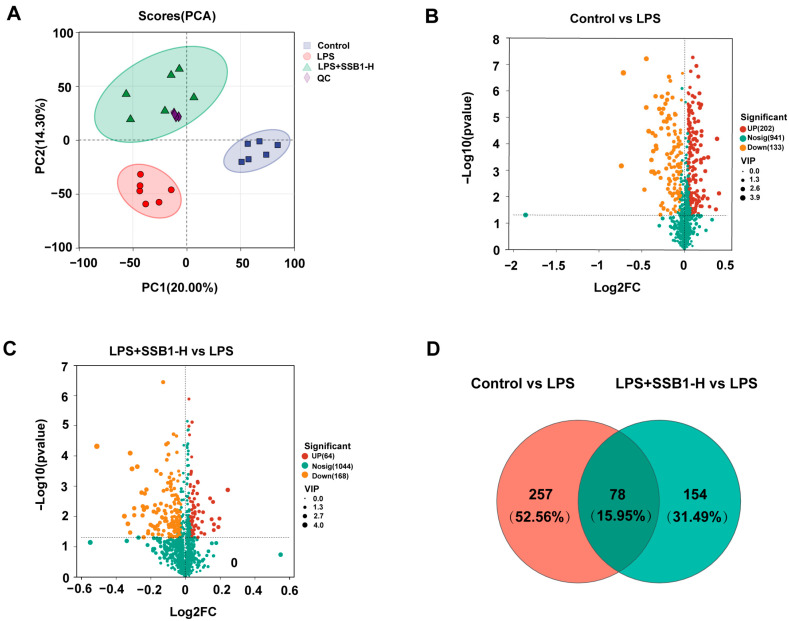
Metabolome analysis of ALI lung tissue after SSB1 treatment. (**A**) PCA of control/LPS/LPS + SSB1-H group in positive and negative ion modes. (**B**,**C**) Volcano plots of control vs. LPS, and LPS + SSB1-H vs. LPS, indicating upregulation (red), downregulation (orange), or no change (green) for different metabolites. (**D**) Changed metabolites of control vs. LPS and LPS + SSB1-H vs. LPS using Venn analysis.

Multivariate analysis of the metabolomic data, including PCA and partial least squares-discriminant analysis (PLS-DA), revealed distinct separations among the control, LPS, and LPS + SSB1-H groups in both positive and negative ion modes (Figure 13A; Supplementary Figure S8A,B). Based on threshold criteria (*p* < 0.05 and VIP > 1), we identified 335 significantly altered metabolites in the LPS group compared to the control (202 upregulated and 133 downregulated). Treatment with SSB1 notably reversed a majority of these changes, resulting in 64 up- and 168 downregulated metabolites relative to the LPS group (Figure 13B,C). Venn analysis further identified a core set of 78 metabolites whose alterations induced by LPS were consistently reversed by SSB1 treatment (Figure 13D). KEGG pathway enrichment analysis of these 78 metabolites suggested that the protective effect of SSB1 is primarily associated with the regulation of glycerophospholipid, linoleic acid, and arachidonic acid metabolism (Supplementary Figure S8C).Detailed change trends of the differential metabolites and their coverage within the top 20 enriched pathways are provided in Supplementary Tables S11 and S12, respectively.

**Figure 14:**
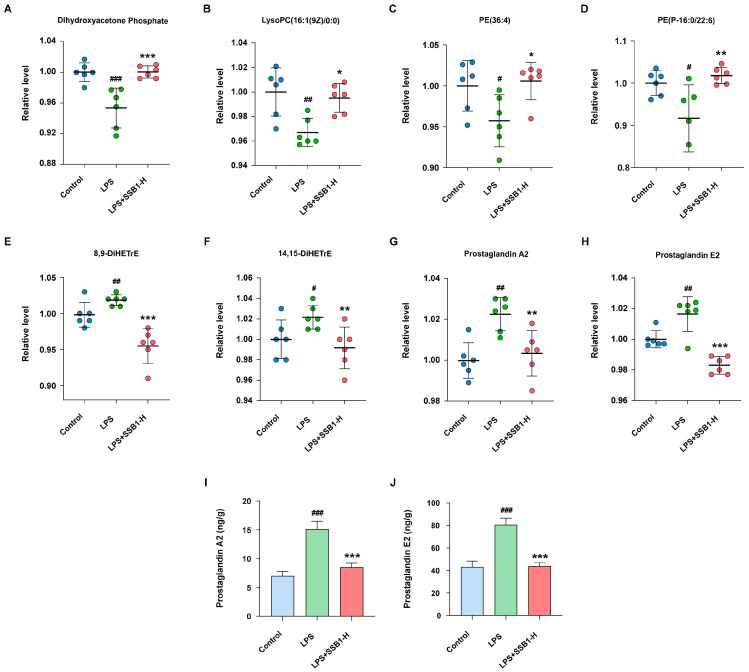
Key metabolites in lung tissue of SSB1 against ALI. (**A**–**D**) Relative levels of four metabolites from arachidonic acid metabolism. (**E**–**H**) Relative levels of four metabolites from glycerophospholipid metabolism. (**I**) Levels of PGA2 and (**J**) PGE2; *n* = 6. **^#^** *p* < 0.05, **^##^** *p* < 0.01, and **^###^** *p* < 0.001 vs. the control group. * *p* < 0.05, ** *p* < 0.01, and *** *p* < 0.001 vs. the LPS group.

We further identified eight key metabolites from glycerophospholipid and arachidonic acid metabolism whose LPS-induced upregulation was reversed by SSB1, including four glycerophospholipid-related (Dihydroxyacetone Phosphate, LysoPC (16:1(9Z)/0:0), PE(36:4), and PE(P-16:0/22:6)) and four arachidonic acid-derived metabolites (8,9-DiHETrE, 14,15-DiHETrE, PGA2, and PGE2) (Figure 14A–H). Given their significant alterations and close association with inflammation, PGA2 and PGE2 were selected for absolute quantification. Targeted metabolomics revealed that LPS induction significantly elevated their levels by 115.6% and 86.8%, respectively, which SSB1 treatment effectively reversed by 43.5% and 45.6% (both *p* < 0.001, Figure 14I,J).

## 4. Discussion

ALI is a life-threatening inflammatory pulmonary syndrome caused by pathogens, physicochemical insults, or immune dysregulation that remains a major global health challenge, posing a significant disease burden due to its high mortality in critically ill patients [5]. The limited efficacy of the current therapeutic options for ALI underscores the urgent need to develop effective treatments and elucidate their mechanisms of action. Hence, natural products with well-defined pharmacological profiles represent promising candidates. Particularly noteworthy is the saponin component SSB1, isolated from *Bupleurum* Radix, which has demonstrated broad pharmacological activities, including anti-inflammatory, antiviral, antitumor, and hepatoprotective effects, positioning it as a compelling candidate for ALI management [14,15]. Given the central role of inflammation in ALI pathogenesis, investigating SSB1’s potential therapeutic utility and its underlying anti-inflammatory mechanisms may provide critical insights for its clinical development in ALI treatment.

This study identified 130 common targets from 206 potential SSB1 targets and 1647 ALI-related targets, defining the core set for SSB1’s intervention in ALI. Construction of an “SSB1–target–ALI” network and PPI analysis has revealed a multi-target mechanism. Subsequent iterative screening based on network topology parameters identified 13 core targets, including IL6, TP53, TNF, STAT3, and IL1B. GO and KEGG enrichment analyses were then performed to elucidate the underlying biology. GO analysis associated SSB1’s effects with processes like the cellular response to LPS and the regulation of inflammation. KEGG analysis highlighted significant enrichment in the NOD-like receptor, IL-17, TNF, and C-type lectin receptor signaling pathways. These pathways function synergistically to co-regulate critical aspects of ALI pathologies, including cell survival, proliferation, inflammation, and angiogenesis [21,22], thereby systematically demonstrating a multi-pathway therapeutic mechanism for SSB1.

To systematically identify the core targets and elucidate the mechanism of SSB1 against ALI, we constructed an “SSB1–target–pathway–ALI” interaction network integrating the screened core pathways, key targets, and active components. Modular topology analysis revealed that SSB1’s therapeutic effects are mediated by the synergistic coordination of multiple biological processes via central hub targets, rather than isolated pathway regulation. The intersection of the network’s top 20 targets with 13 core targets from the PPI network identified 8 key targets: NFKB1, IL1B, TP53, JUN, BCL2, CASP3, TNF, and IL6. Molecular docking demonstrated strong binding affinities (binding energy < −5.0 kcal/mol) between SSB1 and TP53, TNF, JUN, BCL2, IL1B, and IL6. Molecular dynamics simulations further validated the binding stability of these complexes and elucidated their atomic-level conformational dynamics. The distinct dynamic profiles observed among the complexes provide a mechanistic basis for SSB1’s multi-target action.

To independently validate the eight core targets derived from the “SSB1–target–pathway” network, we analyzed consistent DEGs from the GSE2411 and GSE263867 datasets. This analysis identified three overlapping targets, IL1B, TNF, and IL6, indicating their central role in ALI pathogenesis. The consistent upregulation of these targets in both datasets provided transcriptomic evidence for their critical involvement in the pathogenesis of ALI. IL6, a key inflammatory and immunomodulatory cytokine, is activated by IL1B and TNF and participates in the TNF signaling pathway. Animal studies indicate that IL6 knockout or antibody-mediated inhibition alleviates disease progression. For instance, in a rat model of intestinal ischemia–reperfusion-induced ALI, suppression of IL6 significantly improved lung pathology [23]. IL1B, a well-characterized pro-inflammatory cytokine in the IL-1 receptor family, broadly regulates inflammatory and immune responses [24]. Accumulating evidence links dysregulated IL6 and IL1B expression to ALI development, indicating their potential as early diagnostic biomarkers in BALF or serum [25,26]. TNF is a key inflammatory initiator in ALI, rapidly amplifying inflammatory cascades, damaging the alveolar-capillary barrier, and promoting pulmonary edema and respiratory impairment [27]. Collectively, this study demonstrates that SSB1 mitigates ALI-associated inflammation by targeting IL1B, TNF, and IL6, supporting its potential as a therapeutic strategy for ALI.

Infection-induced ALI/ARDS is driven by LPS-induced inflammatory cytokine activation, making non-invasive intranasal or tracheal LPS instillation a well-established model for therapeutic evaluation with reduced procedural complications [28]. Based on our pilot experiments comparing modeling approaches (LPS instillation vs. tracheotomy-based micropump infusion) and doses (1, 3, and 5 mg/kg), we therefore established the ALI model using non-invasive intratracheal instillation of LPS at 3 mg/kg to investigate the pharmacological effects of SSB1. SSB1 treatment significantly improved lung histopathology and suppressed inflammatory responses, as reflected by reduced levels of IL-6, TNF-α, and IL-1β in BALF. Transcriptomic analysis of GEO datasets revealed that IL1B, TNF, and IL6 were consistently elevated, suggesting their role as core inflammatory regulators. This finding was corroborated by our animal experiments, which confirmed upregulated protein expression of these cytokines. The convergence of computational and experimental evidence establishes these factors as central drivers of the ALI inflammatory cascade, validating them as promising therapeutic targets.

Transcriptomics captures system-wide molecular changes, providing a powerful framework for elucidating complex biological pathways and mechanisms of drug action [29]. Our integrated analysis implicated the NOD-like receptor signaling pathway as a critical mechanism for the anti-ALI effects of SSB1. Furthermore, emerging evidence links this pathway to the cGAS-STING signaling axis via regulators such as NLRP6, NOD1/NOD2, and NLRP12, suggesting that SSB1’s therapeutic efficacy may be underpinned by its interaction with this broader network [20]. Given that cGAS-STING inhibition is known to alleviate LPS-induced ALI [30,31], we hypothesized that SSB1 acts through this axis. To test this, we investigated key genes within the cGAS-STING signaling pathway (cGAS, STING1, and TBK1) and core network pharmacology targets (IL1B, TNF, and IL6). LPS stimulation significantly upregulated their mRNA expression, and this effect was consistently reversed by SSB1 treatment. This regulatory pattern aligns with the established literature, wherein these key inflammatory mediators (TNF, IL6, and IL1B) cooperatively amplify inflammation by activating downstream pathways such as MAPK and NF-κB, which are themselves modulated by the cGAS-STING signaling axis [32]. Emerging evidence indicates that the cGAS-STING signaling pathway can indirectly modulate the expression of these cytokines [33,34]. Collectively, our findings are consistent with a model in which the cGAS-STING pathway may transcriptionally regulate IL1B, IL6, and TNF expression through downstream effectors such as NF-κB, thereby promoting inflammatory mediator release and forming a central inflammatory axis. This integrated perspective provides a novel theoretical framework for understanding ALI pathogenesis and warrants further investigation into the development of therapeutic strategies targeting the cGAS-STING pathway and associated inflammatory cascades. It is important to note that our inference regarding cGAS-STING pathway involvement is primarily based on the upregulation of its target genes at the mRNA level. While this provides strong correlative evidence, future studies incorporating protein-level analyses (detection of p-STING, and p-TBK1) and functional genetic or pharmacological perturbations (STING knockdown or inhibition) will be necessary to fully corroborate these findings and establish a direct causal link.

Metabolomics provides comprehensive and dynamic profiling of biomarkers that reflect metabolic changes in response to physiological stimuli, thereby facilitating elucidation of their mechanisms of action within biological systems [35]. KEGG pathway enrichment analysis indicated that the protective effects of SSB1 against ALI are primarily mediated through glycerophospholipid, linoleic acid, and arachidonic acid metabolism. Following LPS challenge, we observed significant upregulation of key metabolites from glycerophospholipid metabolism, including Dihydroxyacetone Phosphate, LysoPC (16:1 (9Z)/0:0), PE(36:4), and PE(P-16:0/22:6), as well as mediators of arachidonic acid metabolism such as 8,9- DiHETrE, 14,15- DiHETrE, Prostaglandin A2, and Prostaglandin E2. SSB1 treatment effectively reversed these metabolic alterations. To further define the role of the arachidonic acid pathway, we conducted absolute quantification of its key metabolites: PGA2 and PGE2. The robust LPS-induced elevation of both metabolites was potently suppressed by SSB1 treatment, which mechanistically implicates the inhibition of this metabolic pathway in the drug’s therapeutic efficacy. Previous studies have established IL1B, TNF, and IL6 as central regulators of the inflammatory cascade in ALI. Mechanistic evidence shows that IL-6 modulates arachidonic acid metabolism through STAT3 and FABP5 signaling, indirectly influencing glycerophospholipid remodeling. TNF regulates arachidonic acid and linoleic acid metabolism via NF-κB and MAPK pathway activation, contributing to inflammation-associated glycerophospholipid metabolic reprogramming [36,37,38]. Although direct evidence for IL1B is less extensive, emerging studies indicate its potential involvement in lipid metabolism through inflammatory signaling [39]. Critically, these genes exhibit cross-regulatory functions in inflammatory responses, metabolic dysregulation, and immune modulation, with particularly pronounced effects on arachidonic acid metabolism. This finding aligns with metabolomic enrichment results and suggests that IL1B, TNF, and IL6 may coordinately regulate multiple interconnected pathways, thereby forming an “inflammation–metabolism” axis that offers new insights into ALI pathogenesis.

This study delineates the therapeutic potential and molecular mechanism of SSB1 against ALI through an integrated multi-omics approach. Network pharmacology predicted 130 shared targets, with enrichment analyses highlighting the NOD-like receptor, IL-17, and TNF signaling pathways. Consolidating the “SSB1–target–pathway–ALI” network and PPI analyses refined a core set of eight targets, including CASP3, NFKB1, TP53, TNF, JUN, BCL2, IL1B, and IL6. Molecular docking and dynamics simulations confirmed stable binding of SSB1 to several core targets, including TP53, TNF, JUN, BCL2, IL1B, and IL6. Transcriptomic profiling of independent GEO datasets and subsequent in vivo validation consistently established IL1B, TNF, and IL6 as crucial inflammatory mediators in ALI, whose aberrant expression was effectively normalized by SSB1. Parallel metabolomics revealed that SSB1’s efficacy involves the reprogramming of glycerophospholipid, linoleic acid, and arachidonic acid metabolism, with IL-6 and TNF contributing to this functional inflammation–metabolism axis. Further transcriptomic investigation pinpointed the NOD-like receptor pathway as a primary mechanism, and positioned the cGAS-STING pathway as an upstream regulator that modulates the expression of IL1B, IL6, and TNF, potentially via transcription factors like NF-κB.

In conclusion, this integrated multi-omics study demonstrates that SSB1 alleviates ALI through multi-targeted synergism, modulating inflammatory responses and restoring metabolic homeostasis. Our findings provide a robust theoretical and experimental foundation for the clinical development of SSB1. It should be noted, however, that the current study has limitations. Primarily, the reliability of network pharmacology predictions is inherently dependent on database completeness and accuracy. Additionally, conclusions derived from this data-driven approach require direct experimental validation through techniques such as Western blotting, gene knockout models, and targeted pharmacological interventions, ultimately progressing to clinical studies. Future work should prioritize these investigations to facilitate the clinical translation of SSB1.

## 5. Conclusions

This study employed an integrated approach that combined network pharmacology, transcriptomics, and metabolomics, supplemented by molecular docking and molecular dynamics simulations, to systematically elucidate the molecular mechanisms underlying the protective effects of SSB1 against ALI. Our findings suggest that SSB1 alleviates ALI by targeting key inflammatory factors, including IL1B, TNF, and IL6, thereby modulating the NOD-like receptor signaling pathway and restoring pulmonary metabolic homeostasis. Together, these results highlight SSB1’s multi-target therapeutic strategy across multiple biological tiers, thereby providing preliminary evidence and laying the foundation for future translational studies.

## Data Availability

The original contributions presented in this study are included in the article/Supplementary Material. Further inquiries can be directed to the corresponding author(s).

## Supplementary Materials

The following supporting information can be downloaded at: https://www.mdpi.com/article/10.3390/metabo15120782/s1, Figure S1: Identification of SSB1 targets; Figure S2: Key targets in the NOD-like receptor signaling pathway; Figure S3: Functional enrichment analysis of key biological themes; Figure S4: The hydrogen bonds and solvent-accessible surface of the complexes. (A) TP53, (B) TNF, (C) JUN, (D) BCL2, (E)IL1B, and (F) IL6; Figure S5: Analysis of ALI-related transcriptomic profiles from GEO datasets. (A) Heatmaps visualizing the expression patterns of the top 50 DEGs in the GSE2411, and (B) GSE263867 datasets; Figure S6: Common DEGs in the GSE2411 and GSE263867 datasets; Figure S7: Transcriptome analysis of ALI lung tissue after SSB1 treatment. (A) TOP 20 enriched GO, and (B) KEGG pathways; Figure S8: Metabolome analysis of ALI lung tissue after SSB1 treatment. (A) Partial least squares-discriminant analysis (PLS-DA) of Control/LPS/LPS + SSB1-H group in positive and negative ion modes. (B) Verification of the PLS-DA model with the 200-fold permutation test. (C) Top 20 enriched KEGG pathways (Metabolite names labeled in red represent up-regulation, while those in blue represent down-regulation). Table S1: Databases used for the target prediction of SSB1; Table S2: Parameters for retrieving ALI-associated targets from public databases; Table S3: Parameters for PPI network construction and analysis; Table S4: Parameters for GO and KEGG enrichment analysis; Table S5: Parameters for Metascape analysis; Table S6: Parameters for molecular docking analysis; Table S7: Transcriptomic data analysis parameters; Table S8: Transcriptome sequencing and analysis parameters; Table S9: Metabolomics analysis parameters; Table S10: Detailed parameters for lipidomics analysis; Table S11: Differential metabolite change trends compared to the model group; Table S12: Metabolite Coverage in the Top 20 Enriched Pathways.

## Abbreviations

The following abbreviations are used in this manuscript:

SSB1	Saikosaponin B1
ALI	Acute lung injury
PPI	Protein–protein interaction
GO	Gene Ontology
KEGG	Kyoto Encyclopedia of Genes and Genomes
LPS	Lipopolysaccharide
BALF	Bronchoalveolar lavage fluid
ELISA	Enzyme-linked immunosorbent assay
H&E	Hematoxylin–eosin
ARDS	Acute respiratory distress syndrome
COVID-19	Coronavirus Disease 2019
TCM	Traditional Chinese Medicine
BR	*Bupleurum* Radix
SSA	Saikosaponin A
SSD	Saikosaponin D
RMSD	Root mean square deviation
RMSF	Root mean square fluctuation
Rg	Radius of gyration
SASA	Solvent-accessible surface area
DXMS	Dexamethasone
RSD	Relative standard deviation
QC	Quality control
PCA	Principal component analysis
PLS-DA	Partial least squares-discriminant analysis
VIP	Variable importance in projection
TPM	Transcripts per million
DEG	Differentially expressed gene
FDR	False discovery rate
SEM	Standard error of the mean
ANOVA	Analysis of variance
BC	Betweenness centrality
BP	Biological process
MF	Molecular function
CC	Cellular component
