# Supplementary material for: Integrative Network Pharmacology and Multi-Omics Analysis Reveal Key Targets and Mechanisms of Saikosaponin B1 Against Acute Lung Injury
Source: Metabolites. 2025 Dec 4;15(12):782. doi: 10.3390/metabo15120782 (PMC12735089; doi:10.3390/metabo15120782)
Supplement: Supplementary file 1 [file metabolites-15-00782-s001.zip › Supplementary Methods.pdf]

Supplementary Methods

2.3 Construction and Analysis of the SSB1-Target-ALI Network

- **Software**: Cytoscape (version 3.9.1)
- **Network Analysis Tool**: Built-in NetworkAnalyzer
- **Primary Topological Parameter**: Node degree

2.7 Network Construction of Target-Pathway Interaction

- **Visualization Software**: Cytoscape (version 3.9.1)
- **Number of Pathways Visualized**: 20
- **Targets Selected for Intersection**: Top 20 by degree centrality

2.9 Molecular Dynamics

- **Simulation software**: GROMACS 2023.4
- **Force field**: AMBER ff14SB
- **Topology generation**: pdb2gmx tool
- **Free energy landscape analysis**: g_sham and xpm2txt modules
- **Visualization**: DuIvyTools 0.6.0
- **Binding free energy calculation**: gmx_MMPBSA with MMPBSA_ana for energy decomposition

2.10 Acquisition and Target Validation from the GEO Database

- **Rationale for Differential Expression Thresholds**: Differential expression thresholds were tailored to the specific technological platform and data characteristics of each dataset. A threshold of $|\log_2FC| > 0.585$ was applied to the microarray data (GSE2411) to account for its inherent technical variability and preserve a broader range of biologically relevant signals. In contrast, a more stringent cutoff of $|\log_2FC| > 1$ was used for the RNA-seq data (GSE263867) to leverage its superior signal-to-noise ratio and focus on the core set of DEGs.

2.11 Animals and Treatments

Clarification on the Dosing Regimen: Prophylactic Administration Strategy

- **Specific Experimental Procedure**: As described in the "Animals and Treatments" section (Section 2.11, Page 5, Lines 199-205), all test compounds (including SSB1 and DXMS) were administered once daily for five consecutive days by oral gavage before LPS challenge. LPS-induced ALI modeling was conducted only after the completion of this 5-day pretreatment period. This design ensured that all drug interventions were completed prior to disease induction, clearly defining the approach as a prophylactic administration protocol.
- **Scientific Rationale for the Study Design**: The main objective of this study was to systematically evaluate the preventive and protective potential of SSB1 against LPS-induced ALI. The decision to adopt a prophylactic dosing strategy was based on the following considerations:

  Targeting Early Pathological Processes: ALI involves a rapid, cascading inflammatory response. We hypothesize that SSB1 may alleviate LPS-triggered inflammatory storm by modulating early signaling pathways (e.g., NF-κB activation) or pre-conditioning the immune system. The pretreatment regimen ensures that the drug reaches effective blood concentrations and engages its targets before injury initiation, which is essential for testing this hypothesis.

  Adherence to Established Research Practices: Prophylactic administration is a well-recognized strategy in preclinical ALI/ARDS research for mechanistic exploration. It is widely used when the objective is to evaluate a compound's potential to prevent or mitigate injury onset, rather than to reverse established damage.

  Foundation for Future Research: This prophylactic study serves as an important proof of concept. Demonstrating significant protective effects under these conditions provides a solid basis for subsequent investigation of the therapeutic efficacy of SSB1 (i.e., post-LPS administration) and helps establish an effective dose range, which is a clear goal of our future work.

Based on these considerations, a prophylactic dosing strategy was deliberately chosen to address the specific scientific question regarding the preventive potential of SSB1.

Additional Clarification on the Control Group Design

- **Drug Administration Perspective**: Establishing an Unbiased Efficacy Benchmark

  To accurately assess drug protection against LPS-induced ALI, all drug treatment groups

received a 5-day pretreatment with drug-containing pure water via gavage. The blank control group was designed to account for two key potential confounders:

Eliminating Effects of the Gavage Procedure: Repeated gavage represents a procedural stress that may modulate the baseline immune status of the animals. By applying the same 5-day pure water gavage protocol, this control group ensures that all groups share a consistent procedural history, thereby neutralizing "gavage stress" as a nonspecific variable in intergroup comparisons.

Confirming Solvent Neutrality: Chemically inert pure water was used as the drug solvent to minimize any potential pharmacological activity of the solvent itself. The "pure water gavage" control provides a pure vehicle reference, ensuring that observed protective effects can be attributed solely to the active pharmaceutical ingredient.

- **Modeling Perspective: Precisely Defining LPS-Specific Injury**

This control group also supports the core objective of delineating LPS-induced injury:

Defining a Healthy Baseline: This group (pure water i.g. + Saline solution i.t.) represents the closest approximation to a healthy state under the experimental conditions, incorporating all pretreatment and procedural steps. Comparison of the LPS model group (pure water i.g.+ 3 mg/kg LPS i.t.) with this baseline allows clear quantification of the pathophysiological changes specifically induced by LPS.

Providing a Robust Reference: As noted by the reviewer, this design offers a "more robust baseline." It addresses the potential limitation of traditional control groups that may overlook the systemic impact of "gavage pretreatment." If drug-treated groups show indicators approaching this robust baseline, conclusions regarding therapeutic efficacy are strengthened.

In summary, this blank control group simultaneously replicates both the "drug administration" pretreatment procedure and the "modeling" instillation operation, serving as an integrated reference benchmark. It ensures that the injurious effects of LPS and the protective effects of the drug can be clearly distinguished within the complex experimental context, thereby enhancing the credibility of the research conclusions.

2.16 Measurement of BALF

- **IL6**: DY406-05; Standard stock concentration: 100ng/mL; detection range: 15.6-1000 pg/mL

- **TNF-α**: DY410-05; Standard stock concentration: 260ng/mL; detection range: 31.2-2000 pg/mL
- **IL-1β**: DY401-05; Standard stock concentration: 110ng/mL; detection range: 15.6-1000 pg/mL

3.8 Molecular Docking

Table 1. Reference Ligands for Core Targets.

Gene	TP53	TNF	JUN	BCL2	IL1B	IL6
Reference Ligand	6VIP	5UUI	5FV8	6GL8	6Y8I	1ALU

Table 2. Molecular docking scores of reference ligands with core targets.

Gene	TP53	TNF	JUN	BCL2	IL1B	IL6
Binding energy (kcal/mol)	-5.73	-3.42	-2.94	-6.50	-3.98	-3.79

Table 3. Molecular docking scores of SSB1 with core targets.

Gene	TP53	TNF	JUN	BCL2	IL1B	IL6
Binding energy (kcal/mol)	-6.36	-5.80	-5.82	-8.18	-6.01	-6.50
