# Supplementary material for: Integrative Network Pharmacology and Multi-Omics Analysis Reveal Key Targets and Mechanisms of Saikosaponin B1 Against Acute Lung Injury
Source: Metabolites. 2025 Dec 4;15(12):782. doi: 10.3390/metabo15120782 (PMC12735089; doi:10.3390/metabo15120782)
Supplement: Supplementary file 1 [file metabolites-15-00782-s001.zip › Supplementary Tables/Supplementary Table S1.pdf]

Supplementary Table S1. Databases used for the target prediction of SSB1.

Database Name	Website URL	Access Date	Primary Purpose
PubChem	https://pubchem.ncbi.nlm.nih.gov	6 July 2025	Retrieve compound structures
ChEMBL	https://www.ebi.ac.uk/chembl/	6 July 2025	Target prediction
TargetNet	http://targetnet.scbdd.com/home/index/	6 July 2025	Target prediction
CTD	http://ctdbase.org/	6 July 2025	Target prediction
BATMAN-TCM	http://bionet.ncpsb.org.cn/batman-tcm/index.php	6 July 2025	Target prediction
SwissTargetPrediction	http://swisstargetprediction.ch/	6 July 2025	Target prediction
PharmMapper	https://www.lilabecust.cn/pharmmapper/index.html	6 July 2025	Target prediction
UniProt	https://www.uniprot.org/	6 July 2025	Protein annotation & filtering
