# Supplementary material for: Integrative Network Pharmacology and Multi-Omics Analysis Reveal Key Targets and Mechanisms of Saikosaponin B1 Against Acute Lung Injury
Source: Metabolites. 2025 Dec 4;15(12):782. doi: 10.3390/metabo15120782 (PMC12735089; doi:10.3390/metabo15120782)
Supplement: Supplementary file 1 [file metabolites-15-00782-s001.zip › Supplementary Tables/Supplementary Table S10.pdf]

Supplementary Table S10. Detailed parameters for lipidomics analysis.

Parameter Category	Specifications
Sample Preparation	**Tissue weight**: 30 mg; **Extraction solvent**: methanol: acetonitrile (5:3, v/v) with 0.001 mg/mL BHT (20 μL/mg); **Homogenization**: vortex 9 cycles (20 s each); **Sonication**: 30 min; **Centrifugation**: 20,000×g, 20 min, 4°C; **Reconstitution**: 100 μL methanol/deuterated solvent (19:1, v/v)
Chromatographic Conditions	**Column**: Waters Acquity UPLC HSS T3 C18 (2.1×100 mm, 1.8 μm); **Mobile phase**: (A) 0.1% formic acid in water, (B) acetonitrile/isopropanol (9:1, v/v); **Gradient**: 0-25 min (25-95% B); **Temperature**: 40°C; **Flow rate**: 0.45 mL/min; **Injection**: 2 μL; **Detection**: PDA full-wavelength scan
Mass Spectrometry Parameters	**Ionization**: ESI negative mode; **Scan mode**: MRM; **Capillary voltage**: 2.5 kV; **Source temperature**: 150°C; **Desolvation temperature**: 600°C; **Cone gas**: 150 L/h; **Desolvation gas**: 1000 L/h
