# Supplementary material for: Integrative Network Pharmacology and Multi-Omics Analysis Reveal Key Targets and Mechanisms of Saikosaponin B1 Against Acute Lung Injury
Source: Metabolites. 2025 Dec 4;15(12):782. doi: 10.3390/metabo15120782 (PMC12735089; doi:10.3390/metabo15120782)
Supplement: Supplementary file 1 [file metabolites-15-00782-s001.zip › Supplementary Tables/Supplementary Table S11.pdf]

Supplementary Table S11. Differential metabolite change trends compared to the model group.

Name	Trend
Hepoxilin A3	down
15-Hete	down
Prostaglandin A2	down
Prostaglandin B2	down
8,9-Dhet	down
14,15-Dihetre	down
9,10-Dihome	down
Vernolic Acid	down
13-Oxoode	down
Phosphodimethylethanolamine	up
Lysopc(16:1(9Z)/0:0)	up
11,12-Epoxyeicosatrienoic Acid	down
20-Hete	down
Dg(18:0/20:4(5Z,8Z,11Z,14Z)/0:0)	down
Gpcho(18:3/22:4)	up
Pe(18:0/20:4(5Z,8Z,11Z,14Z))	up
Pe(36:4)	up
Pe(15:0/18:1(11Z))	down
Pe(P-16:0/22:6)	up
D-3-Phenyllactic Acid	down
3-Hydroxycinnamic Acid	down
Dihydroxyacetone Phosphate	up
Tyramine Glucuronide	down
3-Methoxy-4-Hydroxyphenylglycol Glucuronide	down
Inositol	up
Xanthosine	up

4-O-Alpha-D-Galactopyranuronosyl-D-Galacturonic Acid	up
Phenylpyruvic Acid	down
Ger(D18:1/16:0)	up
L-Threonine	up
L-Phenylalanine	up
L-Histidine	up
D-Xylulose 5-Phosphate	up
Dg(18:0/20:4(5Z,8Z,11Z,14Z)/0:0)	down
