# Supplementary material for: Integrative Network Pharmacology and Multi-Omics Analysis Reveal Key Targets and Mechanisms of Saikosaponin B1 Against Acute Lung Injury
Source: Metabolites. 2025 Dec 4;15(12):782. doi: 10.3390/metabo15120782 (PMC12735089; doi:10.3390/metabo15120782)
Supplement: Supplementary file 1 [file metabolites-15-00782-s001.zip › Supplementary Tables/Supplementary Table S12.pdf]

Supplementary Table S12. Metabolite Coverage in the Top 20 Enriched Pathways.

Rank	Pathway Name	Number of Metabolites Detected	Ratio in study
1	Thyroid hormone synthesis	2	4.08%
2	Aminoacyl-tRNA biosynthesis	3	6.12%
3	Insulin resistance	2	4.08%
4	Protein digestion and absorption	3	6.12%
5	Sphingolipid signaling pathway	2	4.08%
6	D-Amino acid metabolism	4	8.16%
7	AGE-RAGE signaling pathway in diabetic complications	2	4.08%
8	ABC transporters	6	12.24%
9	Pentose and glucuronate interconversions	5	10.20%
10	Adipocytokine signaling pathway	2	4.08%
11	Phenylalanine metabolism	4	8.16%
12	Kaposi sarcoma-associated herpesvirus infection	5	10.20%
13	Neurotrophin signaling pathway	2	4.08%
14	Retrograde endocannabinoid signaling	6	12.24%
15	Vascular smooth muscle contraction	3	6.12%
16	Glycerophospholipid metabolism	8	16.33%
17	Linoleic acid metabolism	4	8.16%
18	Choline metabolism in cancer	3	6.12%
19	Serotonergic synapse	6	12.24%
20	Arachidonic acid metabolism	9	18.37%
