# Supplementary material for: Integrative Network Pharmacology and Multi-Omics Analysis Reveal Key Targets and Mechanisms of Saikosaponin B1 Against Acute Lung Injury
Source: Metabolites. 2025 Dec 4;15(12):782. doi: 10.3390/metabo15120782 (PMC12735089; doi:10.3390/metabo15120782)
Supplement: Supplementary file 1 [file metabolites-15-00782-s001.zip › Supplementary Tables/Supplementary Table S2.pdf]

Supplementary Table S2. Parameters for retrieving ALI-associated targets from public databases.

Database Name	Website URL	Score Threshold	Keyword	Access Date
GeneCards	https://www.genecards.org/	⩾ 30	"acute lung injury"	6 July 2025
HERB	http://herb.ac.cn/	N/A	"acute lung injury"	6 July 2025
OpenTargets	https://www.opentargets.org/	⩾ 0.2	"acute lung injury"	6 July 2025
CTD	http://ctdbase.org/	⩾ 80	"acute lung injury"	6 July 2025
