# Supplementary material for: Integrative Network Pharmacology and Multi-Omics Analysis Reveal Key Targets and Mechanisms of Saikosaponin B1 Against Acute Lung Injury
Source: Metabolites. 2025 Dec 4;15(12):782. doi: 10.3390/metabo15120782 (PMC12735089; doi:10.3390/metabo15120782)
Supplement: Supplementary file 1 [file metabolites-15-00782-s001.zip › Supplementary Tables/Supplementary Table S3.pdf]

**Supplementary Table S3. Parameters for PPI network construction and analysis.**

| Item | Specification |
|---|---|
| Database | STRING (https://string-db.org/) |
| Access Date | 10 July 2025 |
| Species | *Homo sapiens* |
| Interaction Score | $\geqslant$ 0.4 |
| Visualization Software | Cytoscape (version 3.9.1, Cytoscape Consortium, San Diego, CA, USA) |
| Core Target Criteria | Degree, Betweenness Centrality (BC), and Closeness Centrality (CC) all > median value |
