# Supplementary material for: Integrative Network Pharmacology and Multi-Omics Analysis Reveal Key Targets and Mechanisms of Saikosaponin B1 Against Acute Lung Injury
Source: Metabolites. 2025 Dec 4;15(12):782. doi: 10.3390/metabo15120782 (PMC12735089; doi:10.3390/metabo15120782)
Supplement: Supplementary file 1 [file metabolites-15-00782-s001.zip › Supplementary Tables/Supplementary Table S4.pdf]

Supplementary Table S4. Parameters for GO and KEGG enrichment analysis.

Analysis Parameter	Specification
Software Package	clusterProfiler R package (version 4.6.2, School of Public Health, The University of Hong Kong, Hong Kong, China)
Significance Threshold	P value < 0.05
Visualization Terms	Top 15 GO Biological Processes
Visualization Pathways	Top 30 KEGG Pathways
