# Supplementary material for: Integrative Network Pharmacology and Multi-Omics Analysis Reveal Key Targets and Mechanisms of Saikosaponin B1 Against Acute Lung Injury
Source: Metabolites. 2025 Dec 4;15(12):782. doi: 10.3390/metabo15120782 (PMC12735089; doi:10.3390/metabo15120782)
Supplement: Supplementary file 1 [file metabolites-15-00782-s001.zip › Supplementary Tables/Supplementary Table S5.pdf]

Supplementary Table S5. Parameters for Metascape analysis.

Parameter	Specification
Platform	Metascape (https://metascape.org/)
Access Date	12 July 2025
Module Algorithm	MCODE (Molecular Complex Detection)
Degree Cutoff	3
Node Score Cutoff	0.05
K-Core	1.5
Additional Analysis	Transcription factor prediction
