# Supplementary material for: Integrative Network Pharmacology and Multi-Omics Analysis Reveal Key Targets and Mechanisms of Saikosaponin B1 Against Acute Lung Injury
Source: Metabolites. 2025 Dec 4;15(12):782. doi: 10.3390/metabo15120782 (PMC12735089; doi:10.3390/metabo15120782)
Supplement: Supplementary file 1 [file metabolites-15-00782-s001.zip › Supplementary Tables/Supplementary Table S6.pdf]

Supplementary Table S6. Parameters for molecular docking analysis.

Parameter	Specification
SSB1 Structure Source	PubChem (https://pubchem.ncbi.nlm.nih.gov/)
Protein Structure Source	Protein Data Bank (https://www.scsb.org/)
Access Date	16 July 2025
Preparation Software	PyMOL (version 3.1, Schrödinger, LLC, New York, NY, USA), AutoDock Tools (version 1.5.7, The Scripps Research Institute, Olson Laboratory, La Jolla, CA, USA)
Preparation Steps	Remove water molecules, add hydrogens, assign charges
File Format	PDBQT
Docking Software	AutoDock Vina
Visualization Software	PyMOL (version 3.1)
