# Supplementary material for: Integrative Network Pharmacology and Multi-Omics Analysis Reveal Key Targets and Mechanisms of Saikosaponin B1 Against Acute Lung Injury
Source: Metabolites. 2025 Dec 4;15(12):782. doi: 10.3390/metabo15120782 (PMC12735089; doi:10.3390/metabo15120782)
Supplement: Supplementary file 1 [file metabolites-15-00782-s001.zip › Supplementary Tables/Supplementary Table S7.pdf]

Supplementary Table S7. Transcriptomic data analysis parameters.

Parameter	Specification
Analysis Software	R (version 4.4.2)
Normalization Package	limma (version 3.62.2)
GSE2411 Threshold	$\|log_2FC\| > 0.585$ and $P < 0.05$
GSE263867 Threshold	$\|log_2FC\| > 1$ and $P < 0.05$
Visualization Packages	pheatmap (version 1.0.12), ggplot2 (version 3.5.2)
Technical Platforms	GSE2411: microarray; GSE263867: RNA-seq
