# Supplementary material for: Integrative Network Pharmacology and Multi-Omics Analysis Reveal Key Targets and Mechanisms of Saikosaponin B1 Against Acute Lung Injury
Source: Metabolites. 2025 Dec 4;15(12):782. doi: 10.3390/metabo15120782 (PMC12735089; doi:10.3390/metabo15120782)
Supplement: Supplementary file 1 [file metabolites-15-00782-s001.zip › Supplementary Tables/Supplementary Table S8.pdf]

Supplementary Table S8. Transcriptome sequencing and analysis parameters

Category	Specification
RNA Quality Criteria	RIN > 6.5; 260/280 ratio = 1.8-2.2
Sequencing Platform	Illumina NovaSeq X Plus
Sequencing Depth	40 million reads per sample
Quality Control Tools	FastQC, Trimmomatic
Visualization Packages	pheatmap (version 1.0.12), ggplot2 (version 3.5.2)
Alignment Tool	HISAT2
Reference Genome	GRCm39 (mm39) from Ensembl
Quantification Method	RSEM (TPM and raw counts)
Differential Analysis	DESeq2 package
Functional Analysis	GO and KEGG enrichment
