# Supplementary material for: Integrative Network Pharmacology and Multi-Omics Analysis Reveal Key Targets and Mechanisms of Saikosaponin B1 Against Acute Lung Injury
Source: Metabolites. 2025 Dec 4;15(12):782. doi: 10.3390/metabo15120782 (PMC12735089; doi:10.3390/metabo15120782)
Supplement: Supplementary file 1 [file metabolites-15-00782-s001.zip › Supplementary Tables/Supplementary Table S9.pdf]

Supplementary Table S9. Metabolomics analysis parameters.

Parameter Category	Specification
Sample Preparation	40 mg tissue homogenized in 400 μL 80% aqueous methanol
Instrumentation	Thermo UHPLC-Q Exactive HF-X system
Chromatographic Column	ACQUITY HSS T3 (2.1 × 100 mm, 1.8 μm)
Mobile Phase	(A) water with 0.1% formic acid; (B) acetonitrile: isopropanol: water (47.5:47.5:5) with 0.1% formic acid
Mass Range	*m/z* 70-1050
Databases	HMDB (http://www.hmdb.ca/), Metlin (https://metlin.scripps.edu/), and Majorbio (https://cloud.majorbio.com)
QC Criteria	RSD > 30% in QC samples excluded
Statistical Thresholds	VIP > 1, $P < 0.05$
