# Supplementary figures and images for: Integrative Network Pharmacology and Multi-Omics Analysis Reveal Key Targets and Mechanisms of Saikosaponin B1 Against Acute Lung Injury
Source: Metabolites. 2025 Dec 4;15(12):782. doi: 10.3390/metabo15120782 (PMC12735089; doi:10.3390/metabo15120782)

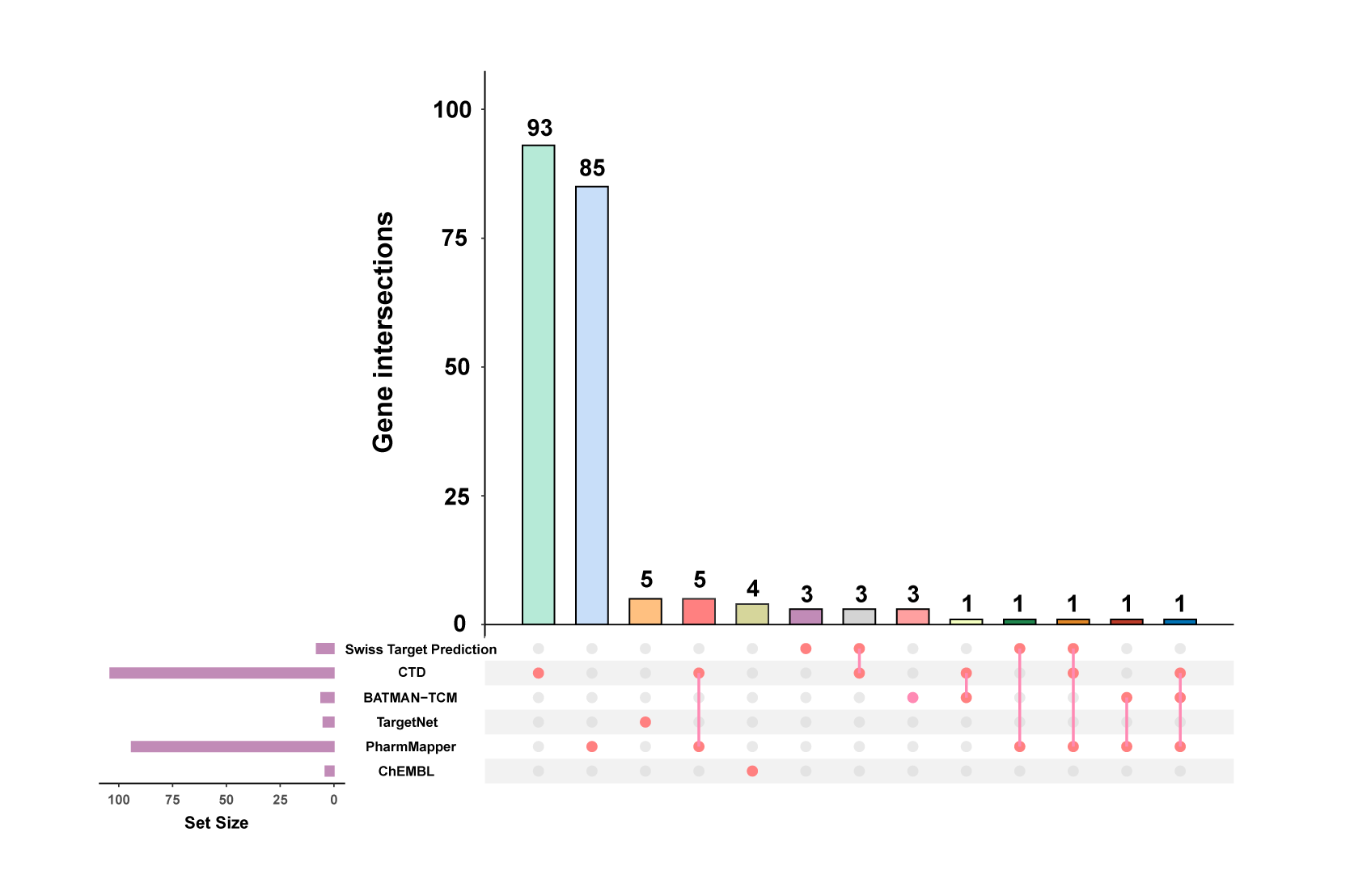

Supplement: Supplementary file 1 [file metabolites-15-00782-s001.zip › Supplementary Figures/Supplementary Figure S1.tif]

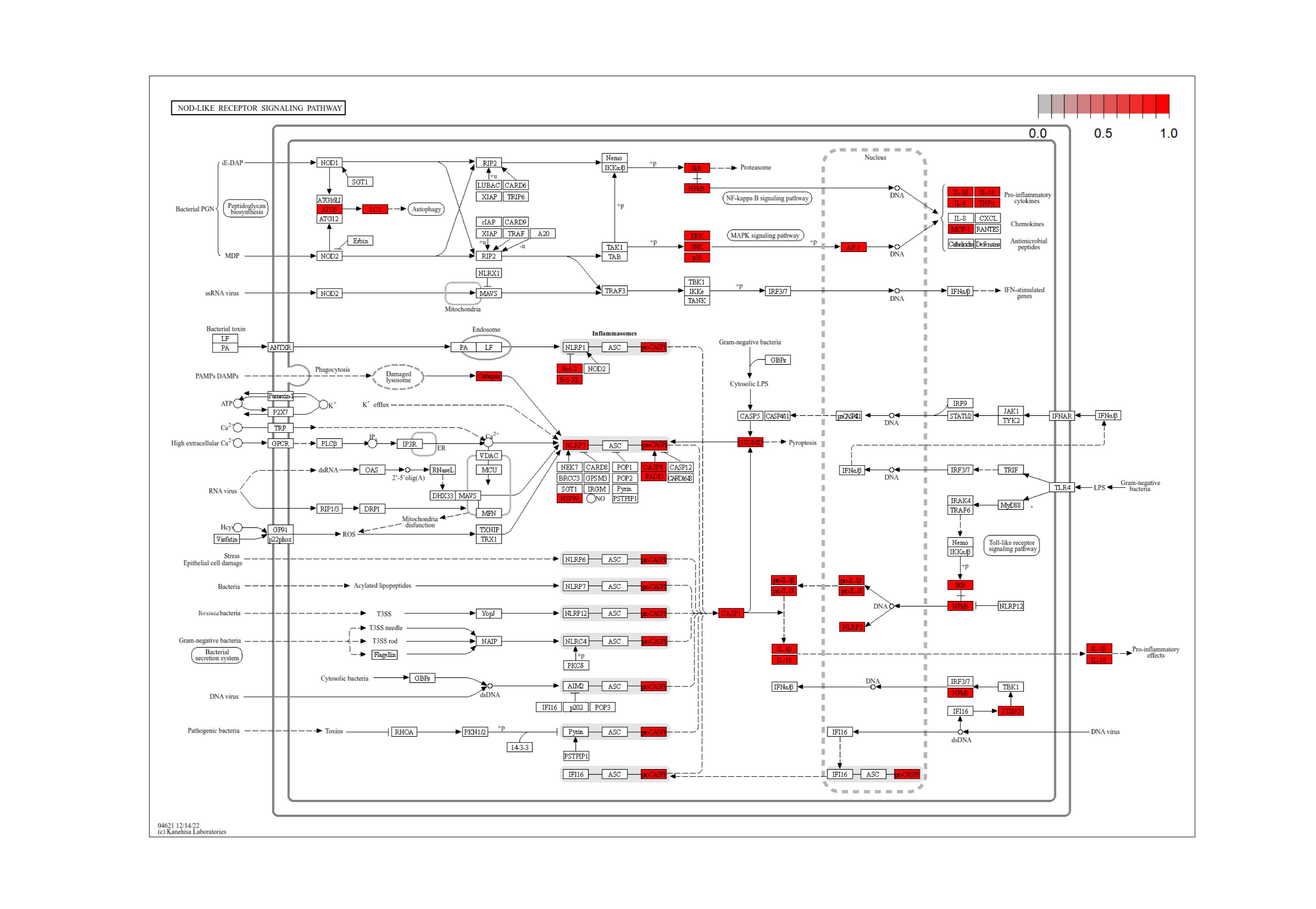

Supplement: Supplementary file 1 [file metabolites-15-00782-s001.zip › Supplementary Figures/Supplementary Figure S2.tif]

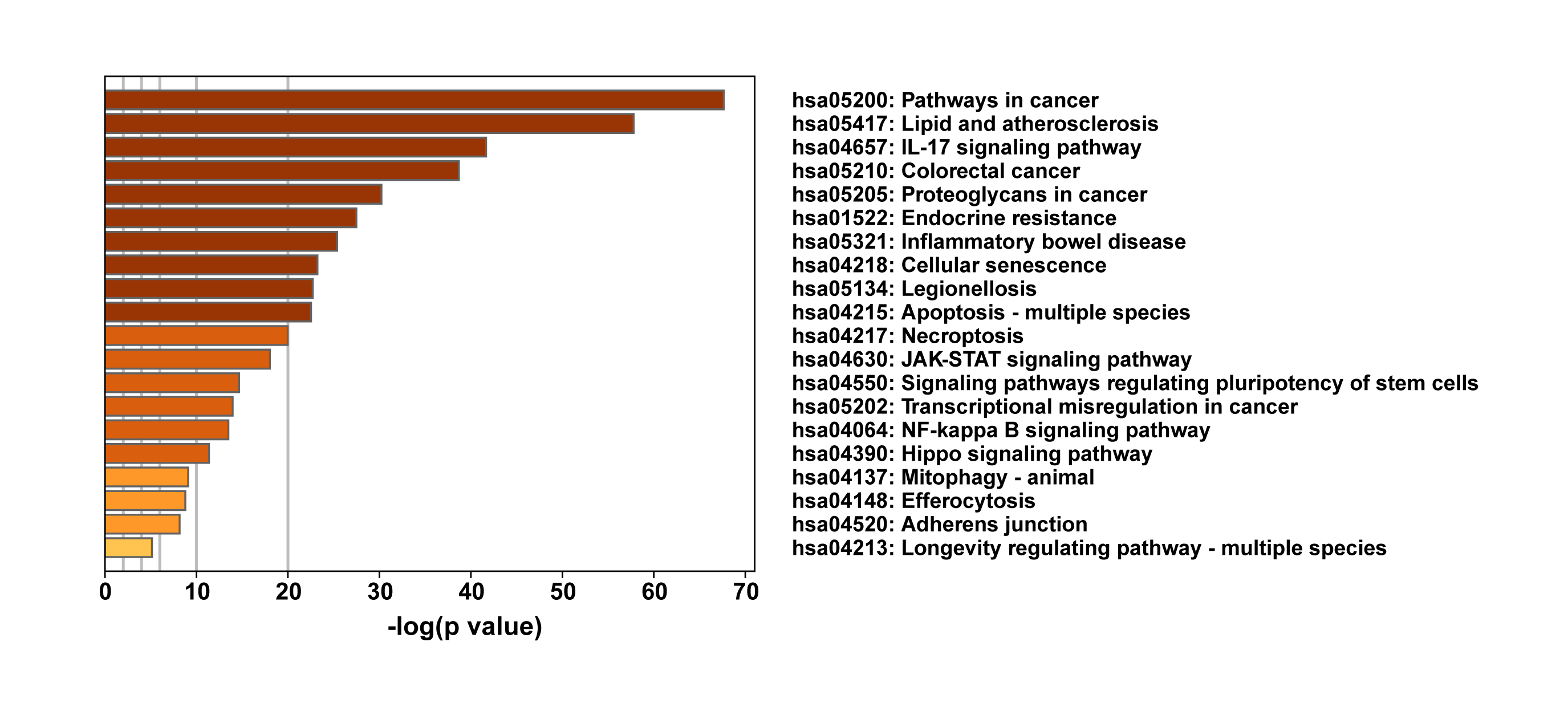

Supplement: Supplementary file 1 [file metabolites-15-00782-s001.zip › Supplementary Figures/Supplementary Figure S3.tif]

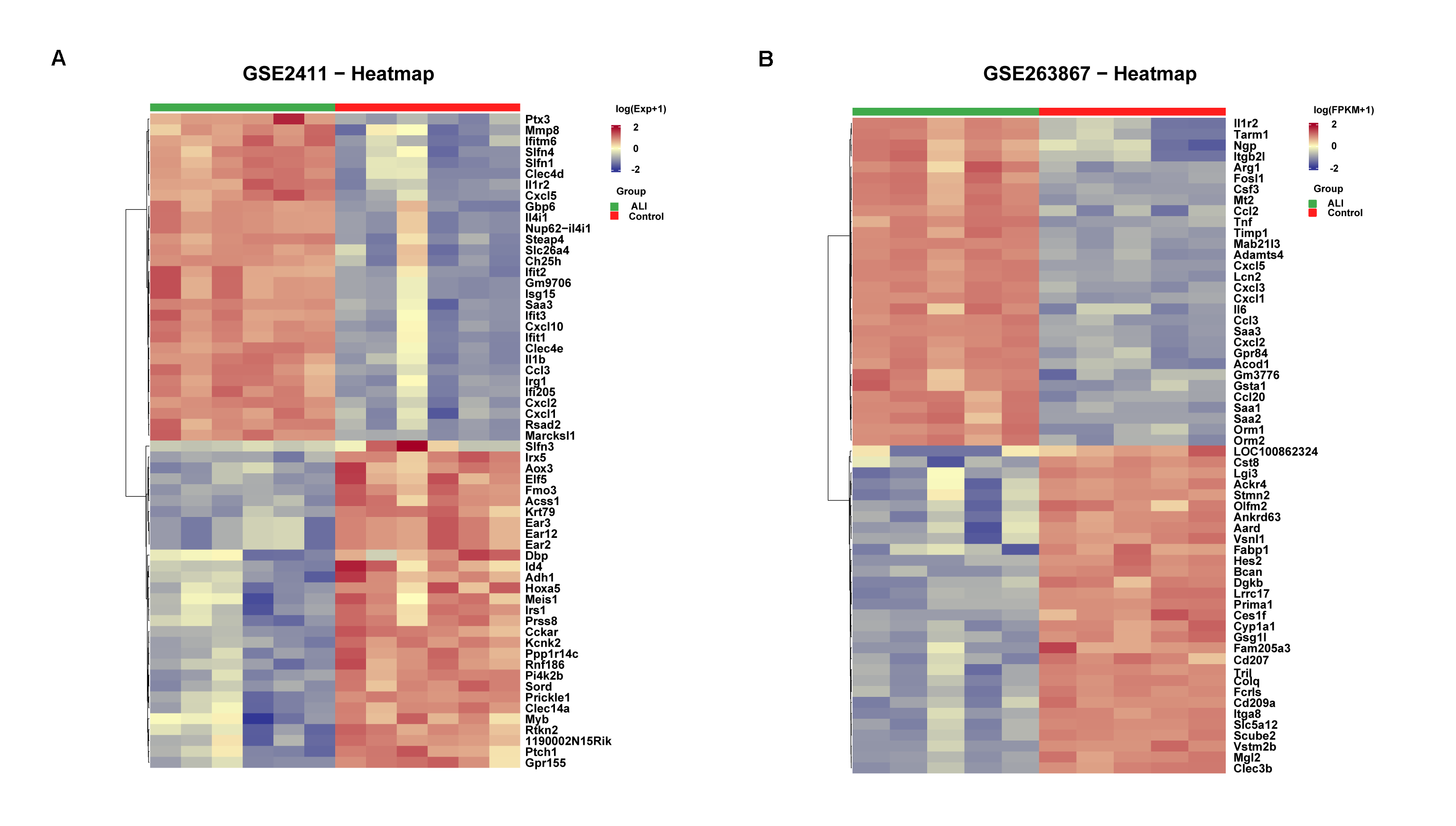

Supplement: Supplementary file 1 [file metabolites-15-00782-s001.zip › Supplementary Figures/Supplementary Figure S5.tif]

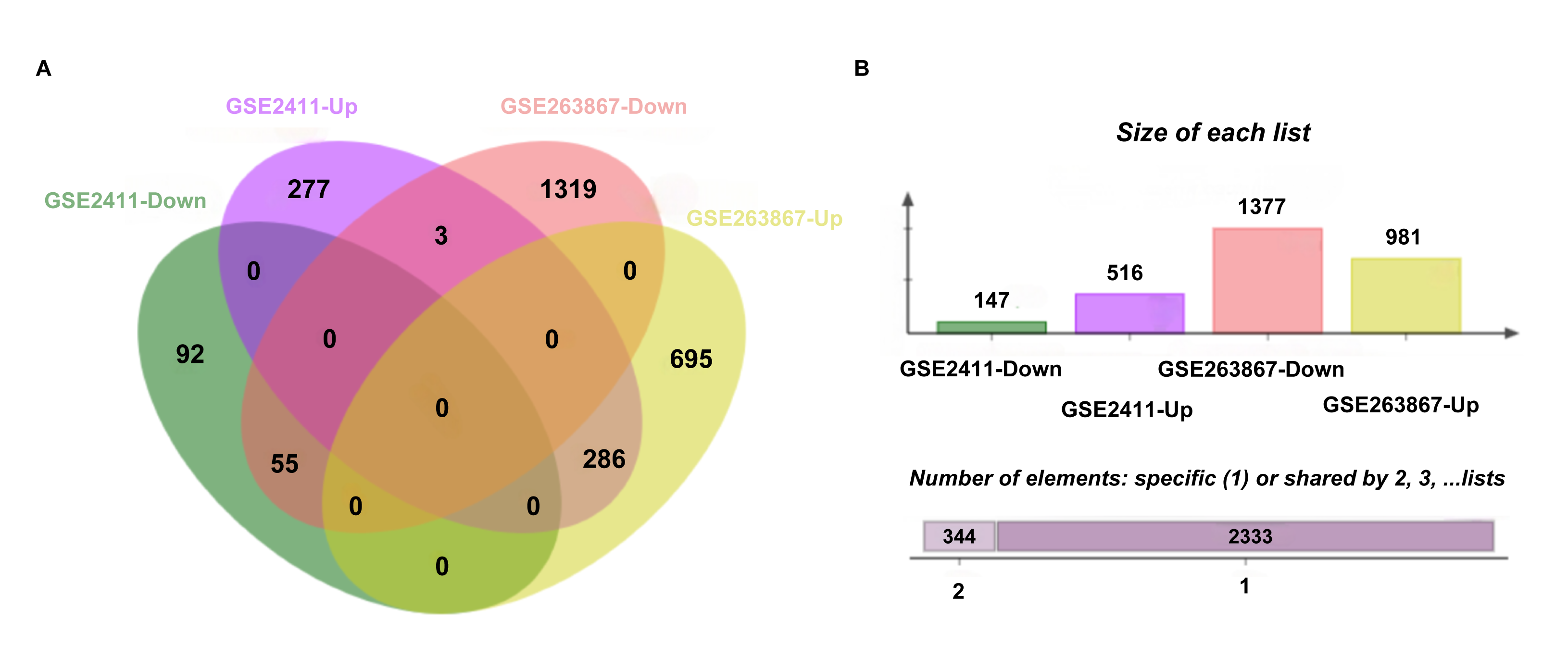

Supplement: Supplementary file 1 [file metabolites-15-00782-s001.zip › Supplementary Figures/Supplementary Figure S6.tif]

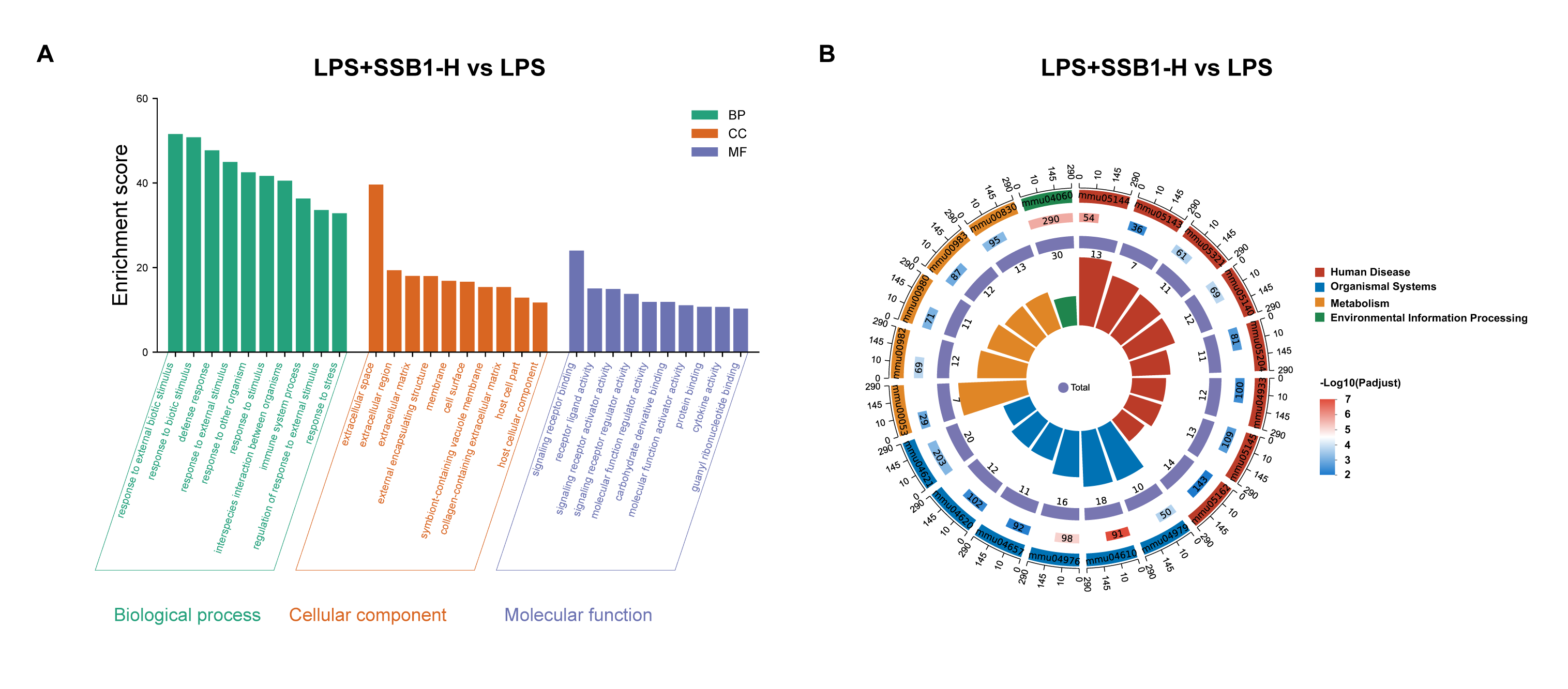

Supplement: Supplementary file 1 [file metabolites-15-00782-s001.zip › Supplementary Figures/Supplementary Figure S7.tif]

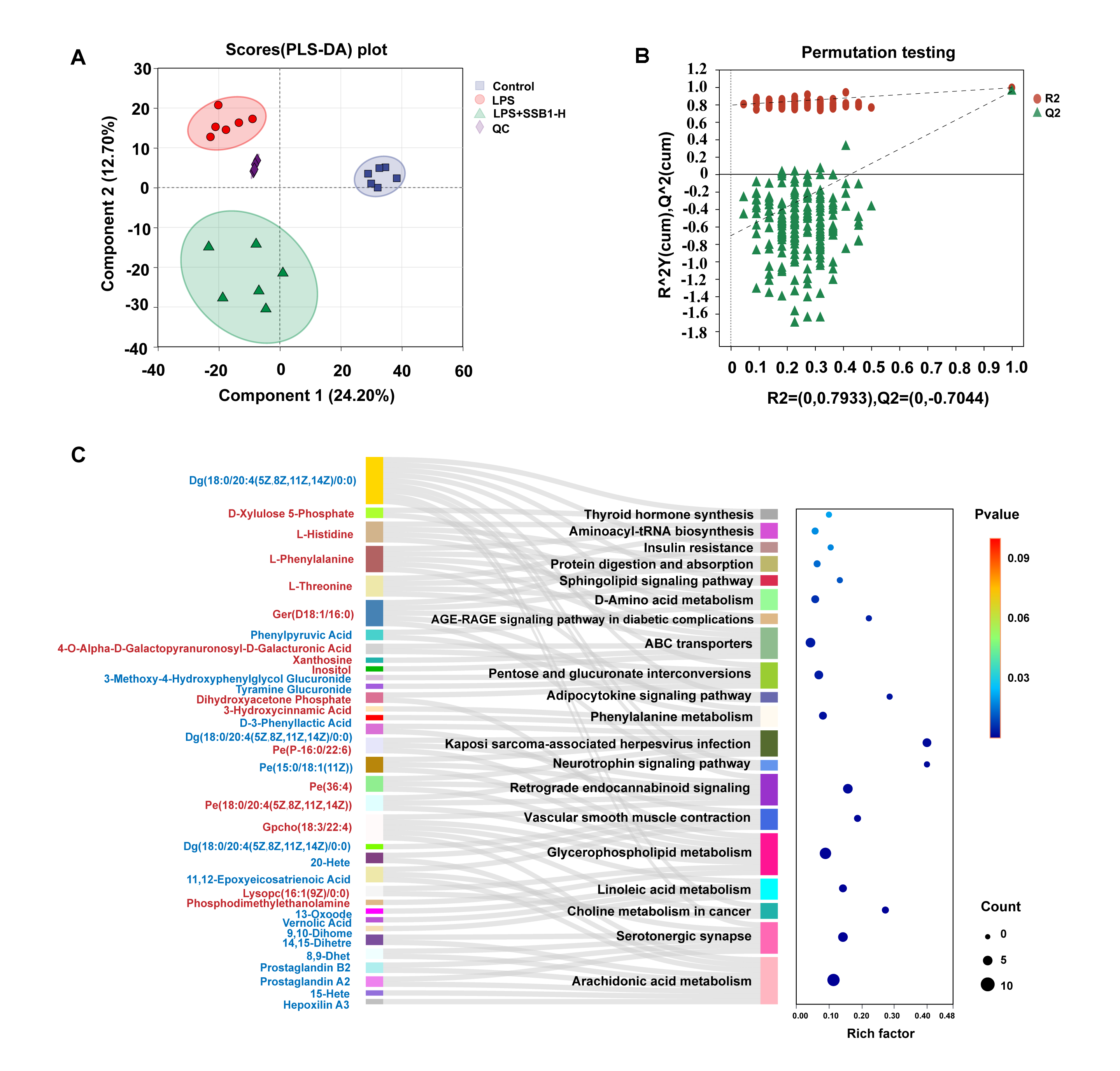

Supplement: Supplementary file 1 [file metabolites-15-00782-s001.zip › Supplementary Figures/Supplementary Figure S8.tif]
